# Endangered *Salares*: micro-disasters in Northern Chile

**DOI:** 10.1080/25729861.2021.1968634

**Published:** 2021-10-07

**Authors:** Cristóbal Bonelli, Cristina Dorador

**Affiliations:** a Anthropology, University of Amsterdam, Amsterdam, Netherlands; b Department of Biotechnology, Universidad de Antofagasta, Antofagasta, Chile

**Keywords:** Atacama Desert, Transdisciplinarity, saltpans, microbial ecologies, extractivism, deep-time, survival, Deserto do Atacama, transdisciplinariedad, salinas, ecologias microbianas, extrativismo, tempo profundo, sobrevivência, Desierto de Atacama, transdisciplinariedad, salares, ecologías microbianas, extractivismo, tiempo-profundo, sobrevivencia

## Abstract

This article emerges from a transdisciplinary collaboration between a micro-biologist and an anthropologist deeply concerned with the protection of endangered *salares* (saltpans) in northern Chile. Our aim is to establish the concept of “micro-disaster” as a tool for examining how extractivism is disrupting *salares* and their “deep-time” microbial ecologies. These ecologies are key for understanding early events on Earth, as their evolution enabled the oxygenation of the planet 2.5 billion years ago and caused the biodiversity explosion. By considering how *being* *human* involves *being* *microorganismal* – and how human time is entangled with microorganismic time –, this article connects neoliberal extractivist history with geo-biological evolutionary history. “Micro-disasters” therefore affect us deeply as complex humans, and oblige us to develop further a planet-centered mode of collaborating, thinking, feeling, and acting. In the context of this special issue on extinction, we insist that concerns over extinction must be considered in continuity with deep-time ecologies. We propose to rethink humans as an “environmentally complex we” simultaneously entangled with historical experiential time and microbial “deep-time.”

## RESUMO

Este artigo surge de uma colaboração transdisciplinar entre uma microbióloga e um antropólogo profundamente preocupados com a proteção de *salares* (salinas) em perigo no norte do Chile. Nosso objetivo é estabelecer o conceito de “microdesastre” como uma ferramenta para examinar como o extrativismo está perturbando os *salares* e suas ecologias microbianas de ‘tempo-profundo’. Essas ecologias são fundamentais para compreender os primeiros eventos na Terra, pois sua evolução permitiu a oxigenação do planeta há 2,5 bilhões de anos e causou a explosão da biodiversidade. Ao considerar como *ser humano* envolve *ser microorganísmico* – e como o tempo humano está enredado com o tempo microorganísmico, este artigo conecta a história extrativista neoliberal com a história evolutiva geo-biológica. Os “microdesastres,” portanto, nos afetam profundamente como humanos complexos, e nos obrigam a desenvolver ainda mais um modo de colaboração, pensamento, sentimento e ação centrado no planeta. No contexto desta edição especial sobre a extinção, insistimos que as preocupações com a extinção devem ser consideradas em continuidade com as ecologias do tempo profundo. Propomos repensar o ser humano como um “nós ambientalmente complexo” simultaneamente emaranhados com o tempo histórico experiencial e o “tempo profundo” microbiano.

## RESUMEN

Este artículo es fruto de la colaboración transdisciplinaria entre una microbióloga y un antropólogo fuertemente preocupados por la protección de salares del norte de Chile que actualmente corren peligro de extinción. Nuestro objetivo es proponer el concepto de “micro-desastre” como herramienta que examina los modos en que el extractivismo está alterando a estos salares y a sus ecologías microbianas de tiempo-profundo. Estas ecologías son clave para entender eventos tempranos en la Tierra, en tanto la evolución de ellas hizo posible la oxigenación del planeta hace 2500 millones de años, causando así la explosión de la biodiversidad. Considerando como el *ser humano* implica un *ser microorganísmico* – y como el tiempo humano está enredado con un tiempo micro-organísmico – este artículo conecta la historia extractivista neoliberal chilena con la historia de la evolución geo-biológica. Por lo tanto, los “micro-desastres” nos afectan fuertemente como humanos complejos, y nos obligan a desarrollar, una y otra vez, un modo de colaborar, pensar, sentir y actuar fuertemente centrado en el planeta. En el contexto de este número especial focalizado en extinción, insistimos en la necesidad de considerar los problemas puestos por la posibilidad de extinción en continuidad con ecologías de tiempo-profundo. Proponemos repensarnos como humanos como un “nosotros ambientalmente complejo,” simultáneamente enredado en el tiempo histórico experiencial y el tiempo microbiano profundo.

“There is at least a risk that there will be no more human history unless humanity undertakes a radical reconsideration of itself.” Félix Guattari, *The Three Ecologies* (2000, p. 68)

This article is the fruit of an ongoing collaboration between the authors of this text. We met while working in northern Chile, our country of origin, in a place that has been deeply marked by extractivist mining, the Atacama Desert. In this environment dominated by extractivism – that is, by the “the appropriation of natural resources in large volumes and/or high intensity, where half or more [of a country’s natural resources] are exported as raw materials, without industrial processing or with limited processing” (Gudynas 2018, 62) – we have cultivated a friendship and learned from each other and from our disciplinary backgrounds and sensibilities. Both of us are driven by the desire to protect various ecosystems and ways of life threatened by extractivist industries, and this article arises from our need to account for how the transformations triggered by the extractivist project have affected us. Moreover, we are driven to consider in this article how our transdisciplinary collaboration and our engagements with the desert’s deep-time microbial habitats have increased our capacity to act, think, and feel (Deleuze 1968).

Cristina is a microbiologist, and Cristóbal is a clinical psychologist and anthropologist. Since we first met in March 2017, we have been experimenting with how to create an alliance with “the power to make concerned people think and act together, enabling each to connect with the ways others come to be concerned” (Stengers 2018, 93). Through our conversations, correspondence, and visits to the *salares*, we have strengthened our willingness to undertake transdisciplinary experimentation to develop new ecological stories and ways of thinking in order to raise others’ level of concern. In the same way, our mutual learning has expanded our shared awareness of the *salares*’ geobiological history, thus cultivating what we call a *deep-time-affection* – a type of affect that, in our case, is mixed together with the ways in which Chile’s neoliberal history has also affected our ways of being, thinking, and feeling. In this sense, and following post-colonial historian Dipesh Chakrabarty (2021), this article connects two temporalities that have affected us *simultaneously*: a human temporality, in particular Chile’s recent neoliberal history, and the deep time of biological evolution. With Chakrabarty, we find it essential to consider the temporality of biological evolution in the time of Anthropocene: “if we do not take into account earth history processes that out-scale our very human sense of time, we do not quite see the depth of the predicament that confronts humans today” (156).

*Salares* are deep time: they are vestiges of ancient paleolakes located in what is now northern Chile, to the north of Argentina and south of Bolivia. Geological and tectonic changes have transformed the lakes into closed evaporite basins in the Andean high plateaus. The *salares* still contain water, either at subterranean or surface levels. The subterranean waters of the *salares* in the hyper-arid Atacama Desert have a high concentration of nitrates (“caliche”), boron, and lithium, and have also been mined for saltpeter since the end of the nineteenth century. In comparison with the *salares*’ deep-time history, which includes the periods of geological formation and biological evolution, the history of the Chilean neoliberal extractivist economy, its historical time, is extremely insignificant. In spite of these disproportionate temporalities, the history of neoliberal extractivism has caused irreparable environmental damage. It is only in recent decades that mining companies have successfully exploited the *salares*’ subterranean waters and brines, an exploitation they typically justify by the current need to implement global energy transitions: the *salares* quench the thirst of lithium for batteries, and molten salts for solar energy storage. This extractive trend is currently increasing. According to a recent report published by the International Energy Agency, there is a looming mismatch between the world’s climate ambitions and the availability of “critical minerals” that are essential to realizing those ambitions. The demand for lithium, for instance, will increase forty-two times if the target is met for zero emissions by 2040.^1^ Given that half of the earth’s identified lithium deposits are found in South America’s arid, otherworldly landscape of high-altitude *salares*, such an increase in demand would be environmentally devastating for the region. Therefore, “Energy transitions” relying on “critical minerals” are – to put it mildly – inconsistent if they do not include a parallel critique of extractivism and its environmental impacts (Babidge 2018; Jerez, Garces, and Torres 2021; Agusdinata et al. 2018; Liu, Agusdinata, and Myint 2019) and if, as we will suggest, they do not seriously consider planetary deep-time microbial ecologies.

*Salares* are, and have been, fundamental to Chile’s economic development from the nineteenth century to the present, an extractivist mode of economic development rooted in “the accelerated extraction of natural resources to satisfy a global demand for minerals and energy and to provide what national governments consider economic growth” (Blaser and de la Cadena 2018, 2; Bebbington 2015; Gudynas 2010; Li 2015; Svampa 2015). This globalizing project, with its unequally distributed economic success, has had high costs. Unique ancient ecosystems, and the irreplicable deep-time interdependent relations that have constituted them for billions of years, have been permanently harmed. For example, the *Salar de Lagunillas* (Tarapacá Region, 3800 MASL) has been irrevocably damaged by over twenty years of water extraction for use in copper production. The *Salar de Punta Negra* (Antofagasta Region, 2945 MASL) has suffered a similar fate. In April 2020, the Chilean State Defense Council accused Minera Escondida/BHP, which has extracted water from Punta Negra since the early 1990s, of “continual, cumulative, permanent, and irreparable environmental damage.”^2^ The Council also filed a lawsuit against one of the globe’s primary copper producers, the state-owned National Copper Corporation of Chile (CODELCO), for “continual, cumulative, permanent, and irreparable” damage caused by thirty-six years of water extraction from the *Salar de Pedernales.*^3^ Yet, shortly before the lawsuit was filed, the government had already agreed on a new forty-seven-year contract for further industrial exploration of the *Salar de Pedernales*.

Other *salares* are currently being considered, enacted, and exploited as resources – water for industrial use, salts for direct extraction – according to this same productivist logic. The waters of the *Salar de Llamara* and the Pampa del Tamarugal – the *salar* that is the focus of this article – are used to establish evaporation ponds for nitrate and potassium salt harvesting. The Pampa del Tamarugal waters, in particular, are overexploited, as their use has increased over 1890% over the past thirty years (Viguier et al. 2019).

We began to carry out joint expeditions to some of the *salares* of northern Chile in 2017. From that point we began to write this article and discuss our concerns during long conversations in Santiago, Amsterdam, San Pedro de Atacama, and Antofagasta, the city where one of us (Cristina) lives, which grew during the saltpeter golden age and continues to be deeply marked by extractivist mining. During these years, our affective engagements with the *salares* have afforded a deep-time awareness of planetary processes deeply disrupted by extractivism; this deep-time awareness, in turn, has nurtured a collaborative affection between us. Moreover, our shared experience of recent Chilean national history has become entangled with this deep-time of evolutionary biological timescales (Davies 2016), and in this article we show how this entanglement took place.

## 1. The Globe and its discontents

**Figure 1.**
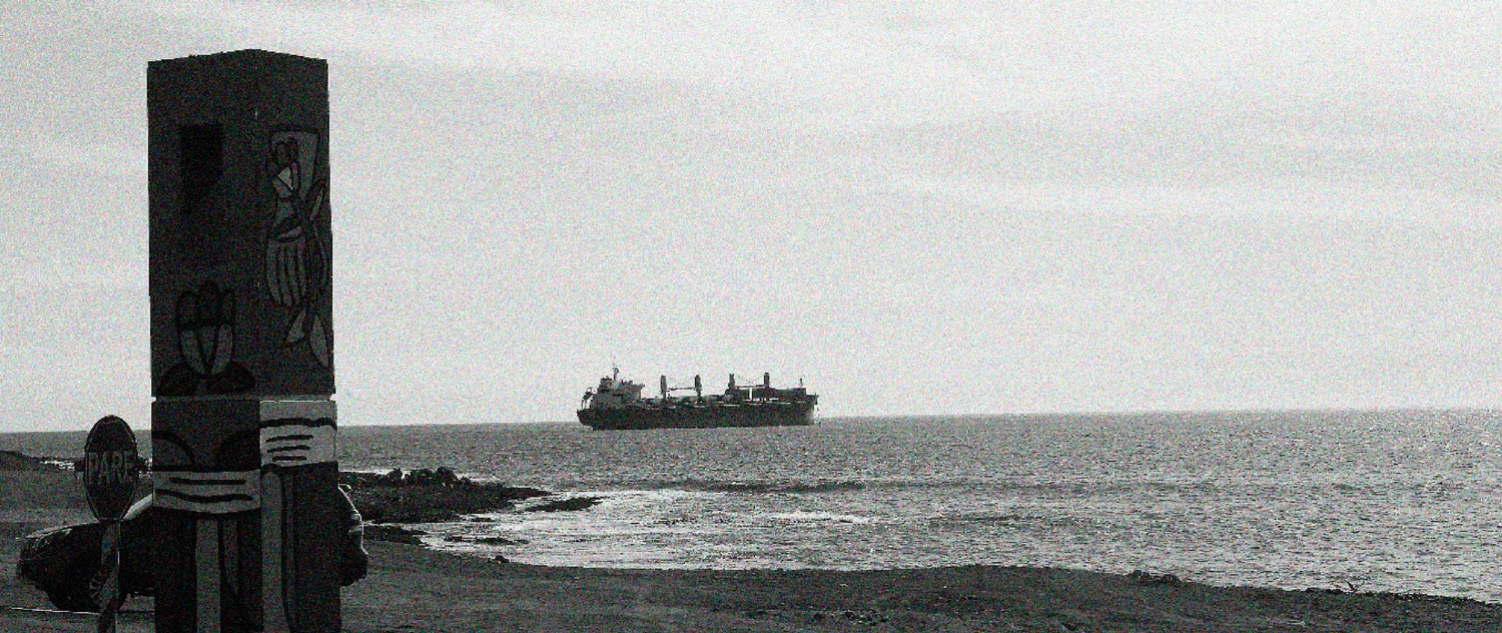
Ship with minerals pulling away from the Antofagasta shore observed from the Memorial dedicated to the victims of the Chilean dictatorship by the Caravan of Death in Antofagasta (credit: Cristóbal Bonelli).

During these years of collaboration and joint research, there is a particular moment we will never forget, a moment that revealed to us, in a clear snapshot, how much work, violence, and destruction the globalized, expansive neoliberal extractivist Chilean project has entailed. This is a snapshot revealing the historical time of the accelerating project of globalization, which in Chile meant the violent imposition of neoliberal policies under the Chilean dictatorship led by Augusto Pinochet (1973–1990) (Figure 1).

We took this picture one afternoon in 2018, after visiting Coloso, a port south of Antofagasta, where huge copper concentrates are dewatered before shipping. On our way back to Antofagasta, we made a stop at the memorial dedicated to all the victims of the Chilean dictatorship by the so-called Caravan of Death (*Caravana de la Muerte*), a Chilean army death squad that flew by helicopter from the south to the north of Chile between September 30 and October 22, 1973.

**Figure 2.**
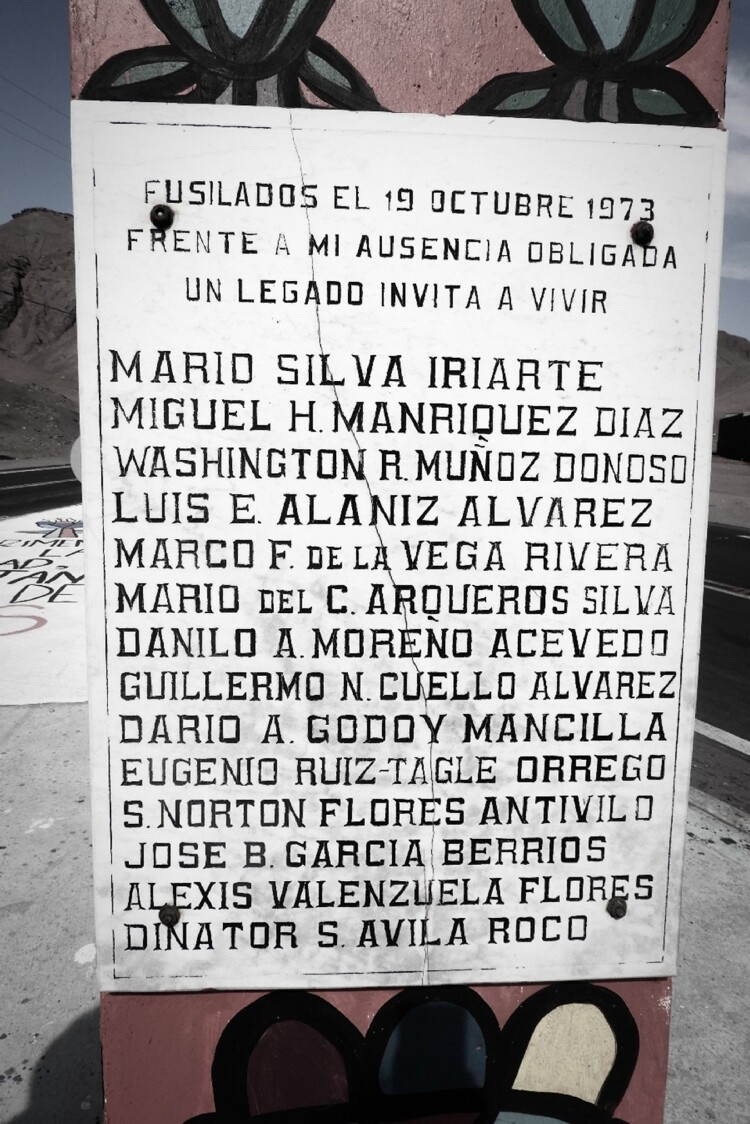
Names of the fourteen people killed by the Caravan of Death on October 19, 1973 (credit: Cristóbal Bonelli).

The photograph shows one of the memorial pillars in front of the sea, and in the sea we can observe a ship pulling away from the shore. Located between the mountains and the sea, the memorial preserves the pain of the assassination of fourteen people carried out at 1:30 am, October 19, 1973, by agents of the military dictatorship. The fourteen killed in Antofagasta had been blindfolded; they were not even allowed to see the stars one last time. We shared a long moment of silence while in the memorial, both of us feeling deeply affected by Chile's history of political violence, by its historical time. (Figure 2).

**Figure 3.**
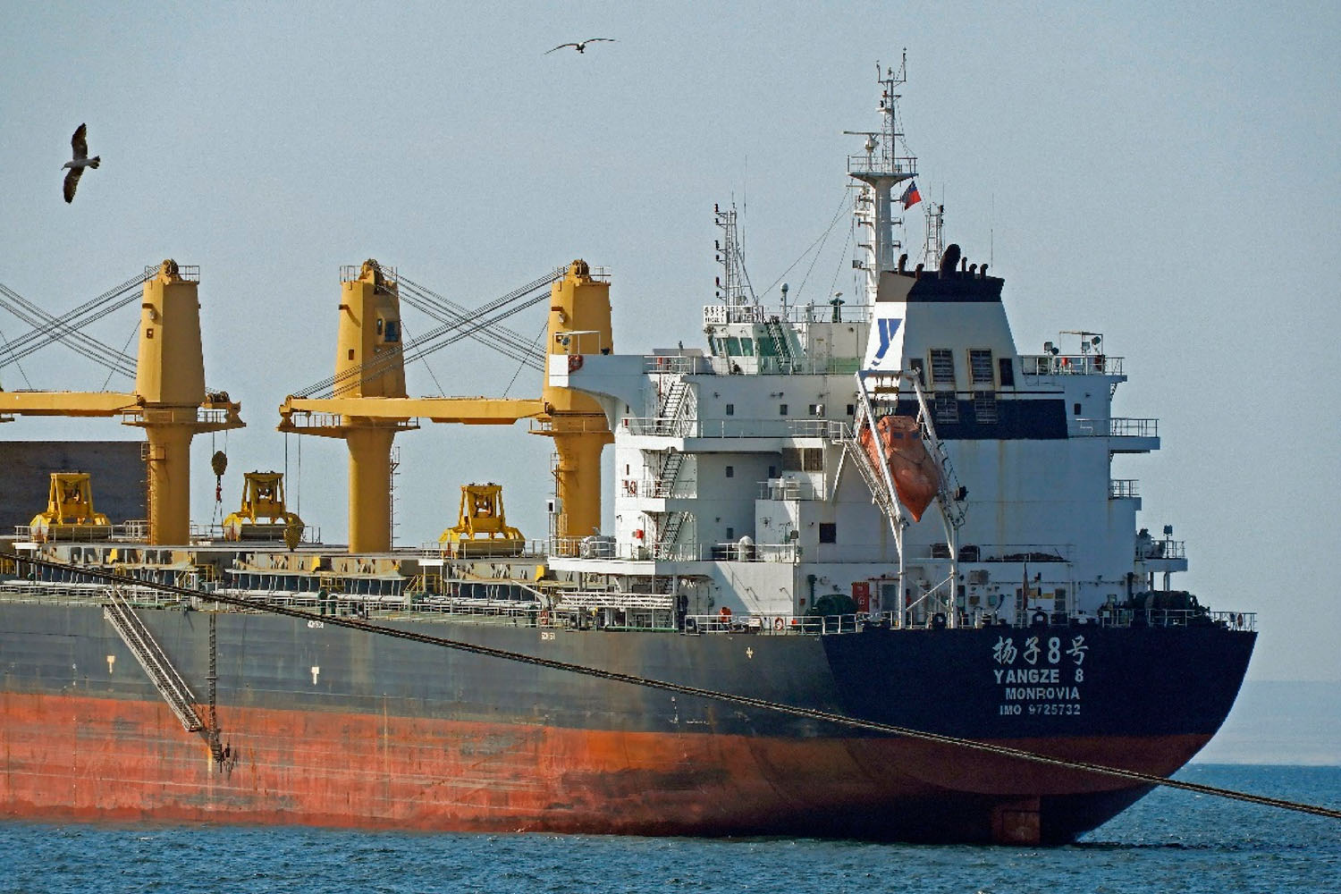
Close up to the ship with a name in Mandarin (credit: Cristóbal Bonelli).

In the ship’s storage holds are some mixture of minerals and water: copper concentrate, to be certain, but likely also lithium carbonate. In the past, ships like this one would have carried saltpeter. The ocean bound freighter is headed to far-away boundaries and industrial plants. Judging from the ship’s name, written in Mandarin, the ship is going to China (Figure 3).

Although we do not have space in this article to give an account of the historical economic relations between China and Chile, it is worth mentioning that during the 1980s the Republic of China – following its own 1978 economic reforms – reactivated commercial relations with the Chilean dictatorship. Chinese leaders were particularly interested in the Chilean economic project, which they saw as a successful economic liberalization without political democratization.^4^ For instance, the military dictatorship privatized Chile’s water and became an international leader in pro-market water resource policies. The Water Code of 1981 introduced a radical separation of land from water, defining the latter as private property free from significant state regulation. The Code triggered equity problems, conflicts among water users, and a severe environmental crisis.^5^

These photos still affect us, reminding us of the moment in which, in a powerful visual way, we were first able to point to and confirm – and experience painfully in our bodies – how Chile’s violent regime was also instituted to facilitate and accelerate extractivism in Chile’s neoliberal contemporary history.^6^ The photo reveals the historical and material connection between, on the one hand, the massacre and brutal population management carried out by the dictatorship and, on the other hand, the establishment of a neoliberal extractive capitalist project, which since 1973 has mined millions of tons of copper, transporting it in ships such as this one, and thereby producing billions of dollars for international capitalists and the national elite.^7^ As such, the photo vividly shows how the mining industry is extractivist in a double sense – it extracts minerals and surplus value in the labor process (Labban 2014; Weinberg 2021). In this article we show how the mining industry also alters the deep-time relational life present in saltpan microbial ecologies and their biological evolutionary history, which is also – as we will show in what follows – , our own biological evolutionary history.^8^ Indeed, in this article we want to show how, in northern Chile, the globe of this globalizing project and the planet, and its bio-geochemical ancestral processes, are indeed connected: “What connects them are the phenomena of modern capitalism … and technology, both global in their reach” (Chakrabarty 2021, 4).

In fact, the pictures enable us to visualize how, when deploying the expansive extractivist, technological, and humanocentric global chain that depends on Chilean minerals, capitalist extractivism violently separates organisms from their environments (Bateson 1972). Indeed, the ship and its Chinese characters show how this capitalist project has expanded through an uprooted and expansive mode of extractivist production, that we could understand, following Tsing (2016), as enacting the ideal of “scalability.”^9^ For Tsing, scalability is:

[The] ability of a project to change scales smoothly without any change in project frames. A scalable business, for example, does not change its organization as it expands. This is possible only if business relations are not transformative, changing the business as new relations are added (…) scalability banishes meaningful diversity, that is, diversity that might change things. (38)

Building upon these ideas, and deeply worried about the current environmental destruction in northern Chile provoked by the extractivist industry, in this article we want to show how the scalability of the expansive neoliberal extractivist project is destroying *salares* while at the same time banishing meaningful microbiological diversity and their relationships of interdependence. In particular, we want to show how, following Shiho Satsuka (2015), this scalability has implied a drawing of one world-making project into another. Indeed, we will show how microbial ecologies of *salares*, and their microbial relations, are certainly un-scalable. Yet, in doing so, we would also like to push forward the relation between this understanding of scalability and the globe of globalization and its extractivist project. We think that this definition of scalability – “the ability of a project to change scales smoothly without any change in project frames” – is not enough to make sense of the temporalities at stake in northern Chile's *salares*, and would benefit, analytically, from explicitly considering how scalability also reveals, following Chakrabarty (2021), the planet:

In thinking historically about humans in an age when intensive capitalist globalization has given rise to the threat of global warming and mass extinction, we need to bring together conceptual categories that we have usually treated in the past as separate and virtually unconnected. We need to connect deep and recorded histories and put geological time and the biological time of evolution in conversation with the time of human history and experience. (7–8)

Therefore, when considering planetary processes, scalability not only refers to the “ability of a project to change scales smoothly without any change in project frames” but also entails the connection between human temporalities and planetary bio-geological processes. Thus, scalability relates different temporal scales, “two different kinds of ‘now-time’ simultaneously” (Chakrabarty 2021; Davies 2016).^10^ In doing so, scalability, we suggest, not only has the potential for decentering the human, but it also offers an opportunity for a radical reconsideration of humanity itself – as Felix Guattari reminds us in the epigraph at the beginning of this article. In fact, in this article we show how scalability alters our own co-existence and continuity with *salares* and, simultaneously, their (our) deep-time microbial ecologies.^11^ In this respect, we hope our work contributes to make visible the invisible *micro-disasters* current extractivist projects entail. With the term *micro-disasters* we want to shed light on a particular kind of slow disaster (Knowles 2014) that connects human time with planetary deep-time, a disaster in which deep-time microbial habitats and relations become vulnerable and disappear. Fully committed to the idea that human thriving relies on an acknowledgment of relations of deep-time interdependence, or as we will see, of intra-dependence, in this article we are interested in showing how *micro-disasters* take place through “densely political relations among many entangled living things – not just microbial – at many scales” (Helmreich 2014). In order to make this point, it is necessary to examine briefly the microbiological practices that have been deployed in the Atacama Desert *salares* in recent decades, practices that have made life visible in a place where it was thought to be absent.

## 2. Microbiology of *salares* in Atacama

For the micro-biological imagination, microorganisms are the most abundant and diverse organisms on the planet. They are found in almost all of the Earth’s environments, including “extreme” ones seemingly hostile to life. The planet’s high microbial diversity strongly depends on microorganisms’ capacity for dispersion and adaptation, as well as on their ability to make connections among themselves and with other species. In other words, the planet is a network of microbial interactions where microorganisms are not only the base and origin of life on Earth and continue to be key to its functioning – but they also constitute us as deep-time human beings.

From a micro-biological perspective, the Atacama Desert was long considered to be devoid of life. Thanks, however, to a “microbial revolution” spurred by huge developments in DNA sequencing, micro-biologists have learned that the desert’s ecosystems are dominated by a high diversity of microbial life. These microorganisms exhibit specific adaptations to deal with high solar radiation, desiccation, exposure to heavy metals, among other conditions, giving important astrobiological clues to the study of the “limits of life” on Earth. Moreover, these microbial communities have a pivotal relevance in deep-time biogeochemical cycles, climate change, and the production of bioactive compounds.

*Salares* are polyextreme ecosystems, which exhibit an extreme dynamic. Water freezes at night, but there are high temperatures and solar radiation during the day. Some sectors of the *salares* are made up of crystalized salts, while others are fresh water. *Salares* receive the highest levels of solar radiation on the planet, as they are hit by an almost unfiltered dosage of sunlight. Microorganisms have somehow adapted to these conditions of excessive energy. One of us, Cristina, had been actively studying the microbiology of northern Chile during the last fifteen years. Her doctoral thesis (Dorador 2007) involved studying samples from the northern Chilean *salares* at the Max Planck Institute of Limnology in Germany. More specifically, Cristina’s doctoral research looked into the functional and taxonomic diversity of the microbial communities of northern Chile’s *salares*, lakes, and wetlands. The samples Cristina studied in these ecosystems enabled her to understand the singularity of different microorganisms, which opened the possibility of coming to know and imagine a microbial reality that exhibits a high level of diversity in each of the *salares*. In fact, today we know that every *salar* on the Altiplano has its own distinctive microbial ancestral digital fingerprint. Each *salar*, moreover, is populated by microbial communities adapted to extreme systems, that is, systems in which life exists under extreme conditions. In functional and evolutionary terms, it may seem like *salares* resemble each other (or that they are functionally redundant), but this resemblance hides an astonishing microbial diversity.

During the period of Cristina’s research, the standard techniques for understanding and studying microorganisms were “culture-independent methods” (based on the study of the DNA or RNA from microbial samples). These methods produced descriptions of microbial communities by sequencing DNA through a process of cloning or direct sequencing of a genetic marker that would give an indication of diversity (e.g. 16S rRNA gene).

In the case of microorganisms from *salares*, working with cultivation-independent methods based on cloning and Sanger sequencing during the 2000s resulted in a limited vision of microbial diversity. More recently, research on environmental DNA has resulted in a higher resolution visualization of the *salares*’ microbial diversity without the need for cultivation. Environments once thought to be devoid of life are now seen as oases of microbial life. For instance, we can now study the microbial communities that live inside of halite in the Atacama Desert.^12^ We can now obtain millions of sequences per sample. When used to study the extreme diversity of the *salares*, these techniques lead to an overwhelming, almost ridiculous conclusion: *diversification tends to infinity*. Although the algorithms used to analyze these sequences diminish the technical issues somewhat, the question remains: what is the minimum unit of an “organism” in the context of a DNA sequence?

This potential bio-logic of the infinite challenges not only the classical taxonomic descriptions used in microbiology to describe and name types of bacteria, but also the definition of species: it makes it difficult to conceptualize a minimum unit of microbial existence. In fact, given that most microorganisms cannot be grown in a laboratory, it has become inappropriate to assign species specificity when one part of a sequence reveals a difference with respect to another part of the sequence.^13^ It is possible that species unity does not even exist at the microbial level. What exists, we could say, is rather sequences of differences that make new multiple interactions and make new differences that make new differences, *ad infinitum* (Bateson 1972).

This microbiological imagination that envisions microbial diversity *ad infinitum* has strong implications when we come to conceive of what gets lost in places like the Atacama desert that are heavily dominated by extractivist industries. What gets lost, we reckon, are deep-time relations – relations not understood as the secondary outcomes of pre-existing, discrete entities, but as a continuous, immanent flux of relations of dependence. In our transdisciplinary conversations, this account of relations strongly resonated with Karen Barad’s (2007) agential realism, which problematizes the usual notion of inter-action, where independently existing entities preexist their acting upon one another:

By contrast, the notion of “intra-action” queers the familiar sense of causality (where one or more causal agents precede and produce an effect), and more generally unsettles the metaphysics of individualism (the belief that there are individually constituted agents or entities, as well as times and places). That is, intra-action goes to the question of the making of differences, of “individuals,” rather than assuming their independent or prior existence. … “[I]ndividuals” only exist within phenomena (particular materialized/materializing relations) in their ongoing iteratively intra-active reconfiguring. (Barad in Kleinman 2012, 77)

Inspired by this understanding of intra-active configurations, of particular interest for our own transdisciplinary collaboration is how developments in the microbiology of *salares* help us understand how relations precede unities. Moreover, considering this microbiological imagination allows us to rethink how extractivist practices – understood as executing a mode of capitalism characterized by an unravelling of “relationships of interdependence” (Stengers 2020a) – alter and, at times, destroy deep-time relations of intradependence on multiple levels through the deliberate production of separated environmental unities susceptible to extraction.

Furthermore, we are aware that the recent developments in microbiology, when examined through STS-anthropological inspired sensibilities, could be considered as part of wider “infra-natures” in the Atacama Desert (Helmreich 2016), an infra-natural desert that has become so scientifically worked upon that it is taken to be nature itself.^14^

However, rather than foregrounding such an STS analysis, and rather than trying to persuade a microbiologist to write as an anthropologist or an anthropologist to become a natural scientist, in this paper we want to experiment with mixing our disciplinary languages and sensibilities as moved by our affective engagements with *infra-natural* *salares* and their microbial ecologies. This transdisciplinary dialogue between us reveals how microbial ecologies afford the generation of a language of alliance potentially capable to unmake dualistic tales of humans versus nonhumans (de la Bellacasa 2017). Indeed, rather than proposing dualistic tales between humans and non-humans, we offer an experimental way of reconsidering the human as constituted by deep-time relations and intra-actions that precede unities (Haraway 2016). In doing so, we are inspired by Lynn Margulis and Fester’s work (1991), which shows how novel biological forms evolve, not only from Darwinian “descent with modification,” but also through the symbiotic, intimate fusion of different types of cells and organisms. Margulis’ perspective underscores the impossibility of conceptualizing unique entities isolated from the world. Margulis’ endosymbiosis theory is key to understanding the origin of eukaryotes. The theory demonstrates how the fusion of bacterial and archaean cells generates a new type of cell, the eukaryotic cell, which takes its nucleus from a bacteria (Alphaproteobacteria) and its cell structure from an archaea receptor. The same process occurs with eukaryotic vegetable cells, whose nuclei are derived from cyanobacteria. Biological intraactions and changing planetary conditions favored the evolution of new species, generating the massive development of biodiversity in the Cambrian Period (the “Cambrian explosion”). This is why, “from the long view of geological time, symbioses are like flashes of evolutionary lighting” (Margulis 1998, 8).

This theory has strongly influenced the most recent work of the biologist and feminist theorist Donna Haraway (2016). Haraway invites us to survive on a wounded planet by conceptualizing and creating space for a “sympoetic imagination,” an imagination that enables us to think freely about how unities do not precede their relations. (Haraway 2016; McFall-Ngai 2017).^15^ In what follows we will show how our own “sympoetic imagination” was nurtured and expanded through and by our affective engagements with *salares* and the ways we have been affected by their deep-time microbial ecologies. These experiential engagements with *salares* have increased our capacity to feel the deep-time stories of our damaged planet, whose ancient geobiological processes are being disrupted by “separatist” extractivist operations and their subject-object grammars. In the following we take a closer look at a particular event we witnessed in May 2017.

## 3. *Salar de Llamara*’s deep affections (Figure 4)

Among the fieldtrips across different *salares* in northern Chile that we have carried out since we first met, we would like to focus on one particular event that took place when we visited the *Salar de Llamara*, a very fragile fossil saline ecosystem situated at the heart of the Atacama Desert, in the Pampa del Tamarugal, where the saltpeter extraction industry developed in the early twentieth century. The name “Tamarugo” comes from a native desert tree that draws water from underground aquifers. The tree is highly adapted to the region’s environmental conditions. Nodes in the Tamarugo’s roots have bacteria that attach to nitrogen – this symbiosis enables the tree to survive in nutrient-poor soil. The Pampa del Tamarugal watershed used to have forests of Tamarugo trees, but their use as an energy source by extractivist industries have made them almost extinct (Castro 2020). Lumber and carbon from Tamarugo trees were used to generate the primary source of energy for the silver industry, saltpeter, as well as to create energy for the Tarapacá *guano* ports in the nineteenth and twentieth centuries (Figure 5).

**Figure 4.**
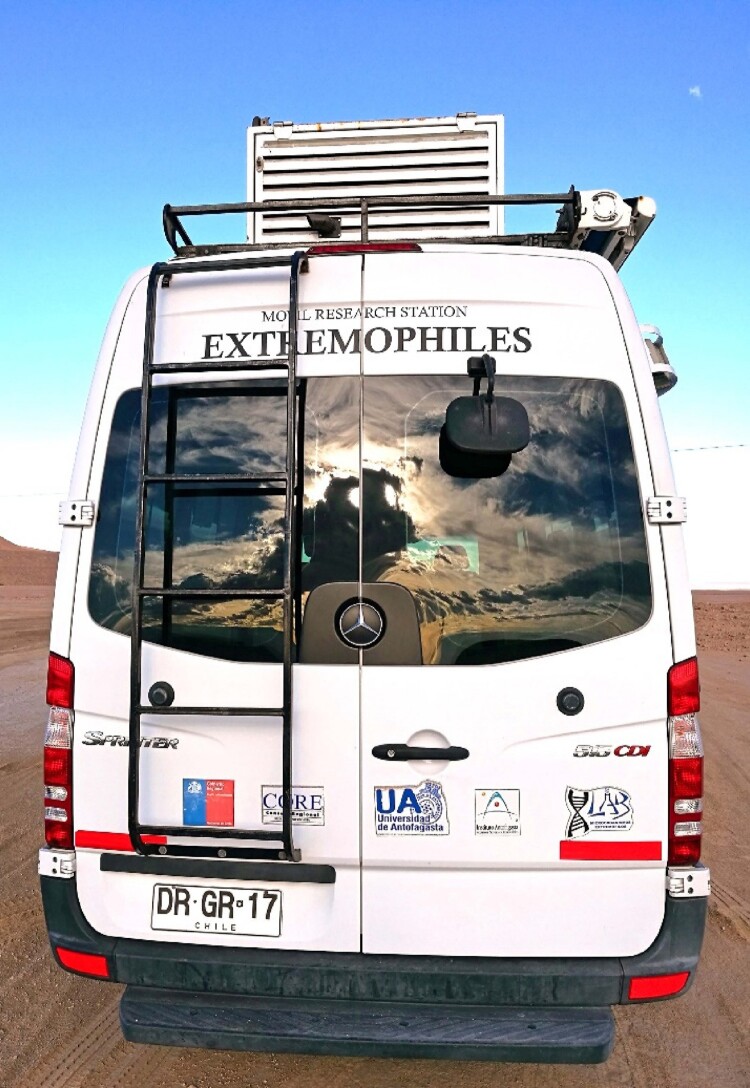
Extremophiles' mobile research station (credit: Cristóbal Bonelli).

**Figure 5.**
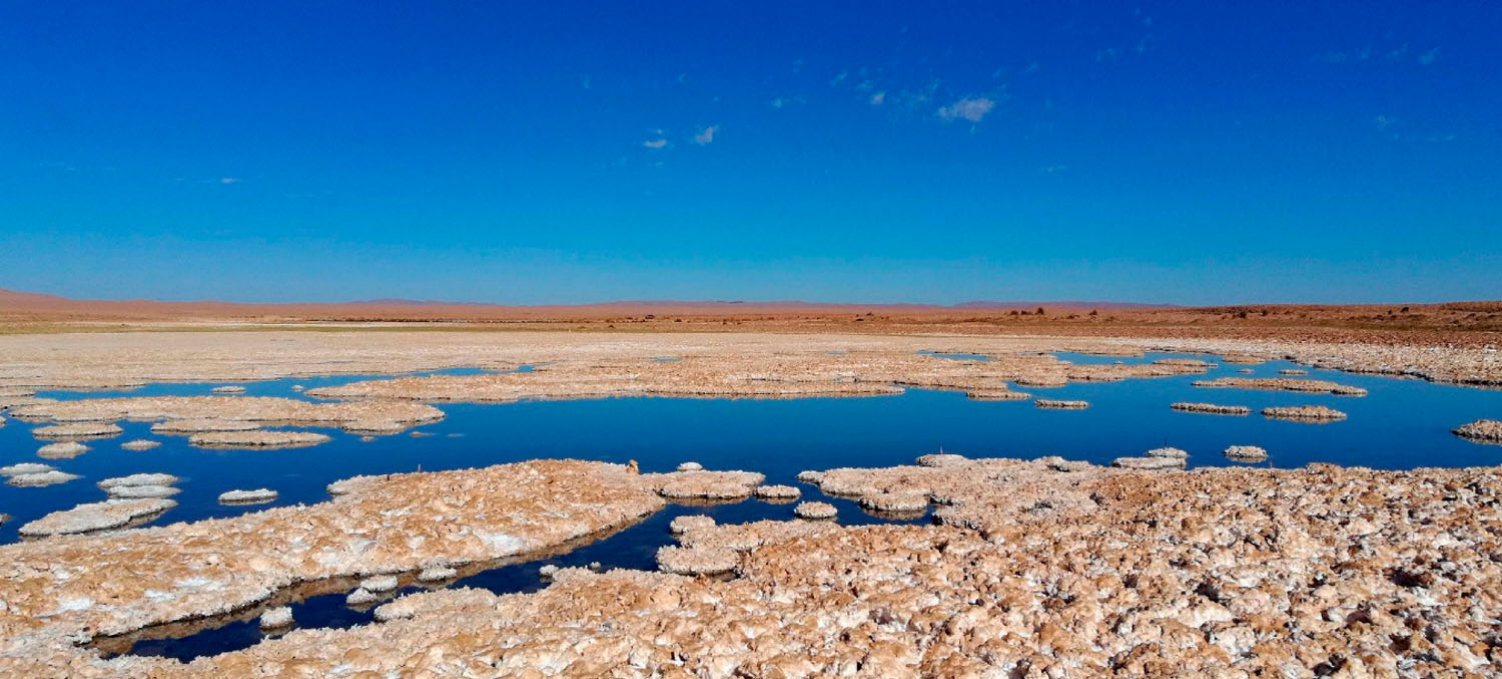
*Salar de Llamara* (credit: Cristóbal Bonelli).

The *Salar de Llamara* is located within the Pampa del Tamarugal National Reserve (created in 1987). Previous research in microbiology has focused on the *salar*’s gypsum formations, which have structural similarities to stromatolites (Demergasso et al. 2003). The pan’s ancient microbial metabolisms – oxidation of carbon monoxide (King 2015) – have also drawn the attention of astrobiologists, and its microbial mats are considered key for understanding early events on Earth, such as the Great Oxidation Event, the moment in which bacteria (specifically, cyanobacteria) began to produce oxygen (Gutiérrez-Preciado et al. 2018).

The *Salar de Llamara* is unique in that it not only allows research into biological evolutionary deep-time and progress, into the genesis of life, but also into *evolution toward death*. *Salar de Llamara* is accordingly called a “terminal system,” a system in a state of stagnation, in which microbial life can be studied for how it stays alive, how it resists death. It is a system that recapitulates the opening chapters of life on the planet, like a summary of the beginning that comes at the end. The very low levels of rainfall in this part of the Atacama Desert make evaporation a continual, negative process: the salar loses more rain than it takes in, making for an extremely fragile ecosystem in which even small changes are significant. Llamara's fragility enables us to imagine the future disappearance of other bodies of water that are being gradually affected by processes of hyper-salinization and evaporation. In what follows we show how we have been strongly affected by this biological evolutive deep-time in our engagements with the *salar*, thus allowing us to cultivate a kind of deep-time affection that tends to be kept separated from experiential human time (Chakrabarty 2021).

**Figure 6.**
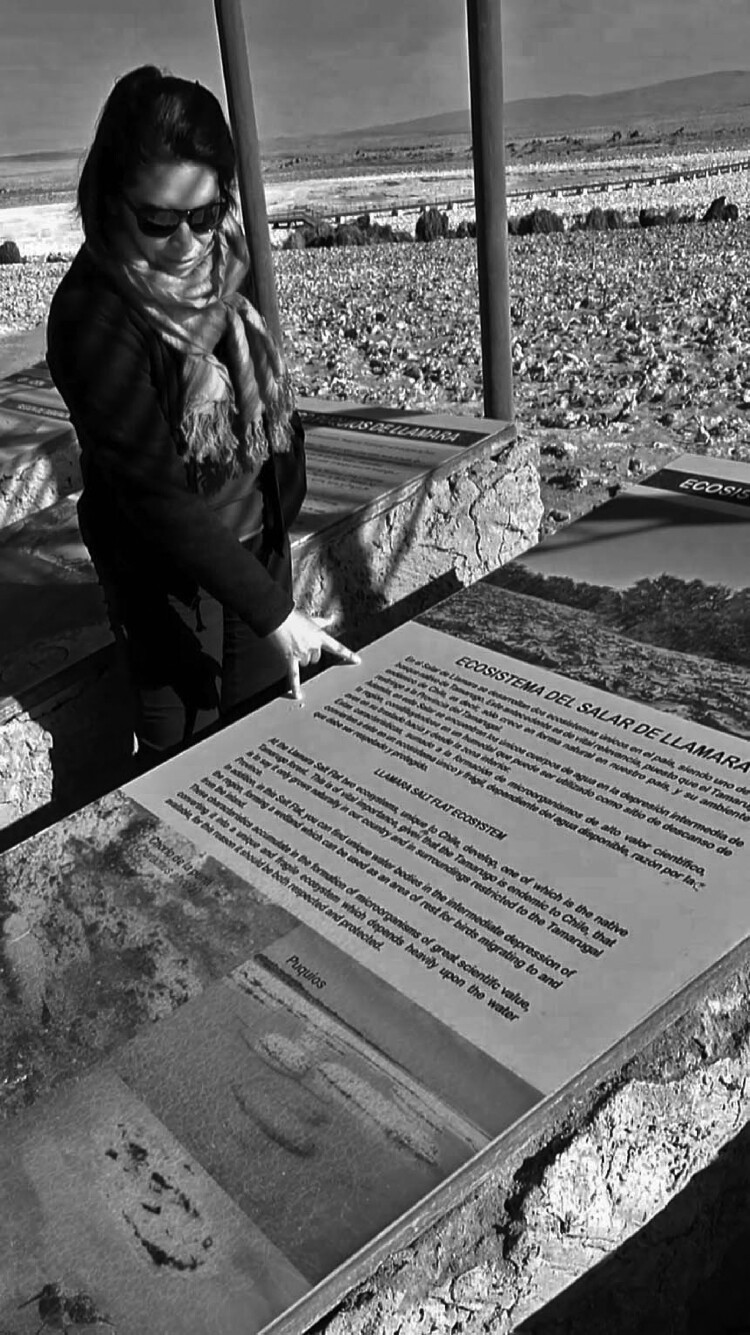
Cristina reading signs with information about microorganisms (credit: Cristóbal Bonelli).

**Figure 7.**
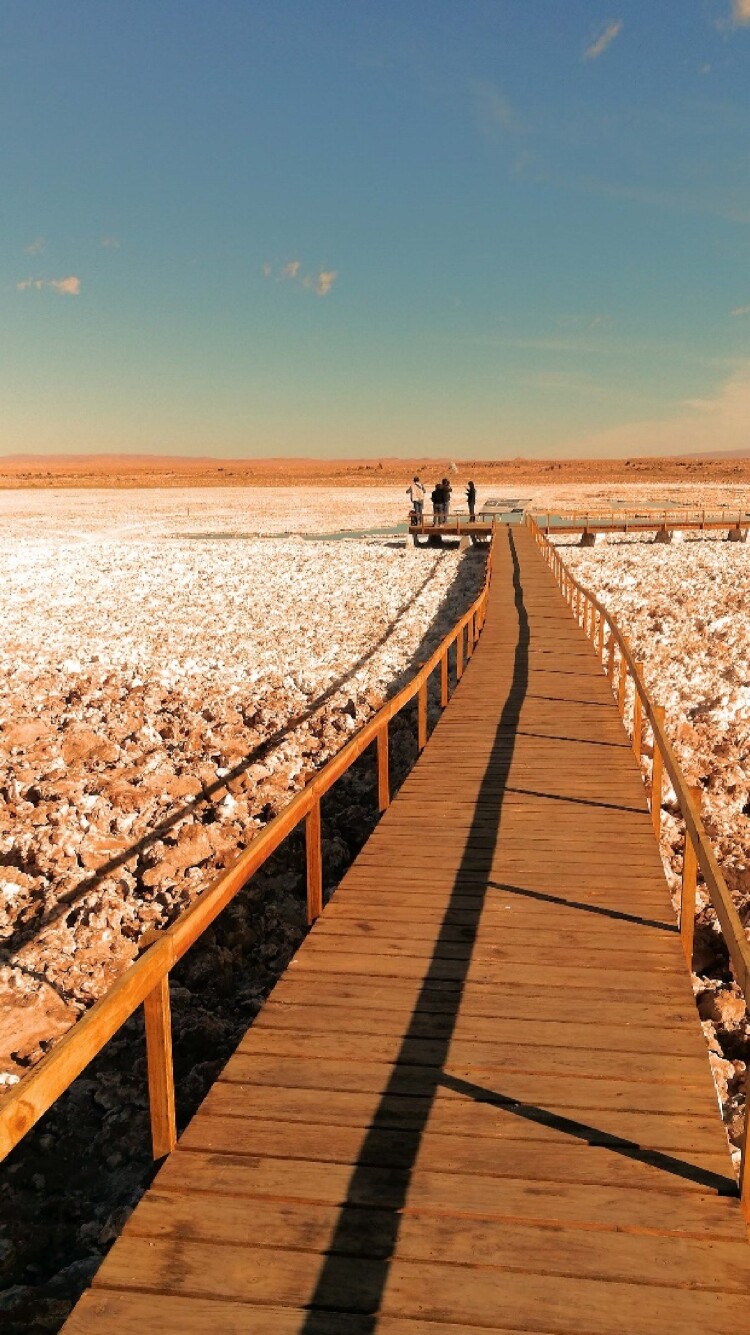
Access platform that allows tourists to get closer to the salar waters (credit: Cristóbal Bonelli).

Immediately after we reached *Salar de Llamara* in May 2017, Cristóbal noticed with surprise how Cristina and the other microbiologists – who were familiar with the area – were emotionally shocked by the visible transformation of the *salar*’s surroundings: the mining company working in that area had built a network of signs containing information for the public about microbial life, as well as an access platform that allows tourists to get closer to the *salar* waters (Figures 6 and 7).

“This is an outrage! This is an outrage,” was the phrase repeated over and over by different members of the team upon seeing the signs. Cristina, reading the signs, responded with sarcasm and anger to emphasize how absurd it was for them to mention “conservation” in a place destined for mining activities that exploited the water from the *salar* and its aquifer.

When we reached the platform overlooking the *salar* pools, we read information about microorganisms that was provided to fulfill the requirements of Chilean environmental law (maintenance of an “environmental baseline”). In spite of the outrage that the signs and the platform provoked, the microbiologists felt that the inclusion of this information was important, since the law in fact traditionally only covers flora and fauna, not microorganisms. Nevertheless, the overall effect of these transformations was to confirm the team’s sense of the futility of environmental impact studies – the signs drew on studies that, according to different members of the team, had been conducted by consultants selected and paid for by the mining company.^16^

**Figure 8.**
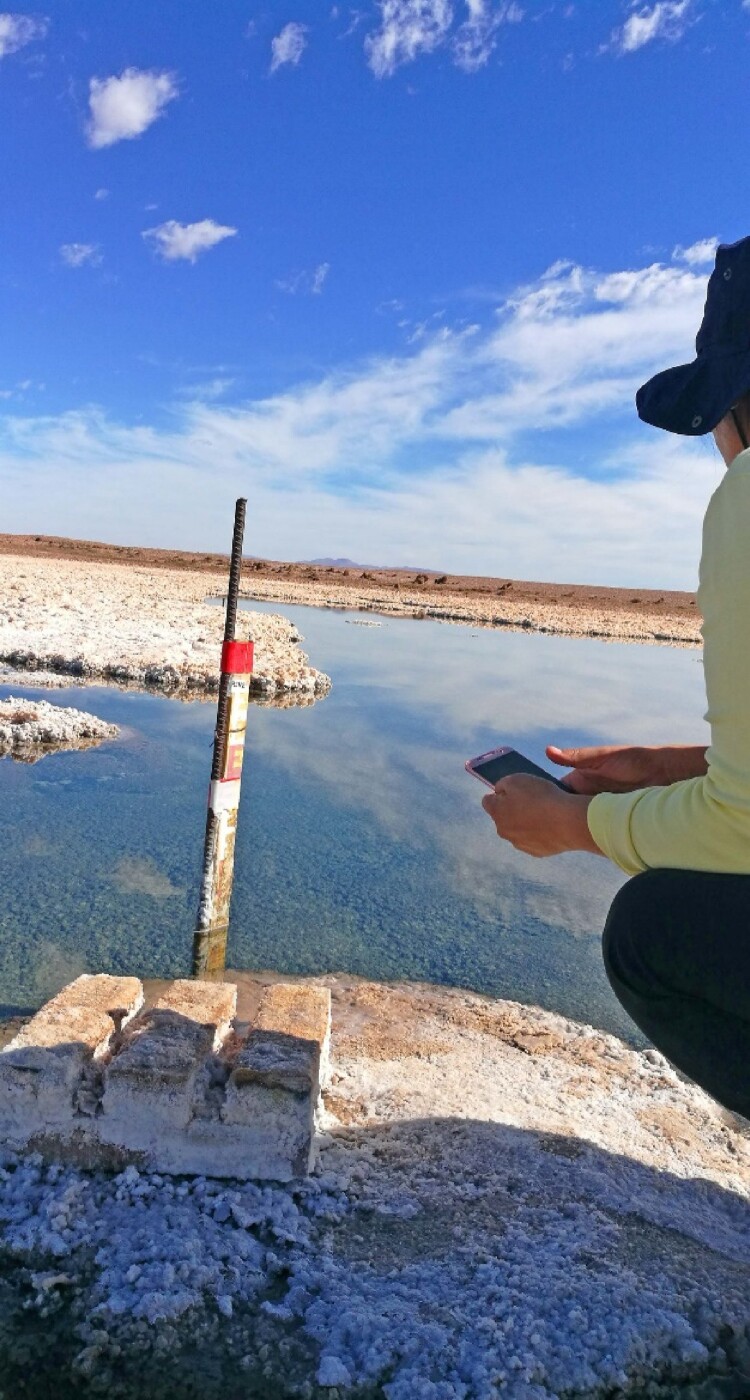
Stick used to measure water levels (credit: Cristóbal Bonelli).

Cristina and her team were sad, angry and frustrated – feelings that Cristóbal began to share as he picked up on the importance of what they were witnessing. The team saw these cosmetic changes as violent interventions in the microbial life of the *salar*, and as a strategy of the mining company to superficially decorate the environmental damage they had produced. To make things worse, when we got closer to the *salar* we noticed that there was less water than usual. Our sense of outrage only grew when we approached the small red and white stick used to measure water levels, an object that is commonly photographed by administrators during environmental management inspections (Figure 8).

According to one of the microbiologists,

The Regional Council of Tarapacá and the community filed a lawsuit [against the mining company] in response to the impact [of its work] on the *salar*. Now [the company] is required by law to maintain the water level in order to preserve the *salar*’s natural springs. The company prefers to submerge the springs so that they won’t suck in all the water and dry everything out … .

You can tell how high the water was if you see that there was salt here – here where there is white stuff. The water reached this level and inundated the area … . Someone stopped it here, but this is a fresh mark – you can tell because there is still salt here, it will fall away later. It reached here because the area was inundated. This is where [inspectors] come to see if [the mining company] is really injecting water [into the *salar*], to see if the water level is OK … . They do all of this at night: they pump out water and take a picture to prove that they are effectively controlling the water level. Then, when the water is really low, they open the spigots and pump in fresh water though these big black hoses … . It’s as if you had a tank of saltwater fish, and every time the water got low you filled the tank back up with fresh water … it’s obvious that the fish aren’t going to respond well, that they’re going to die. All the microorganisms here are adapted to super salty water!

**Figure 9.**
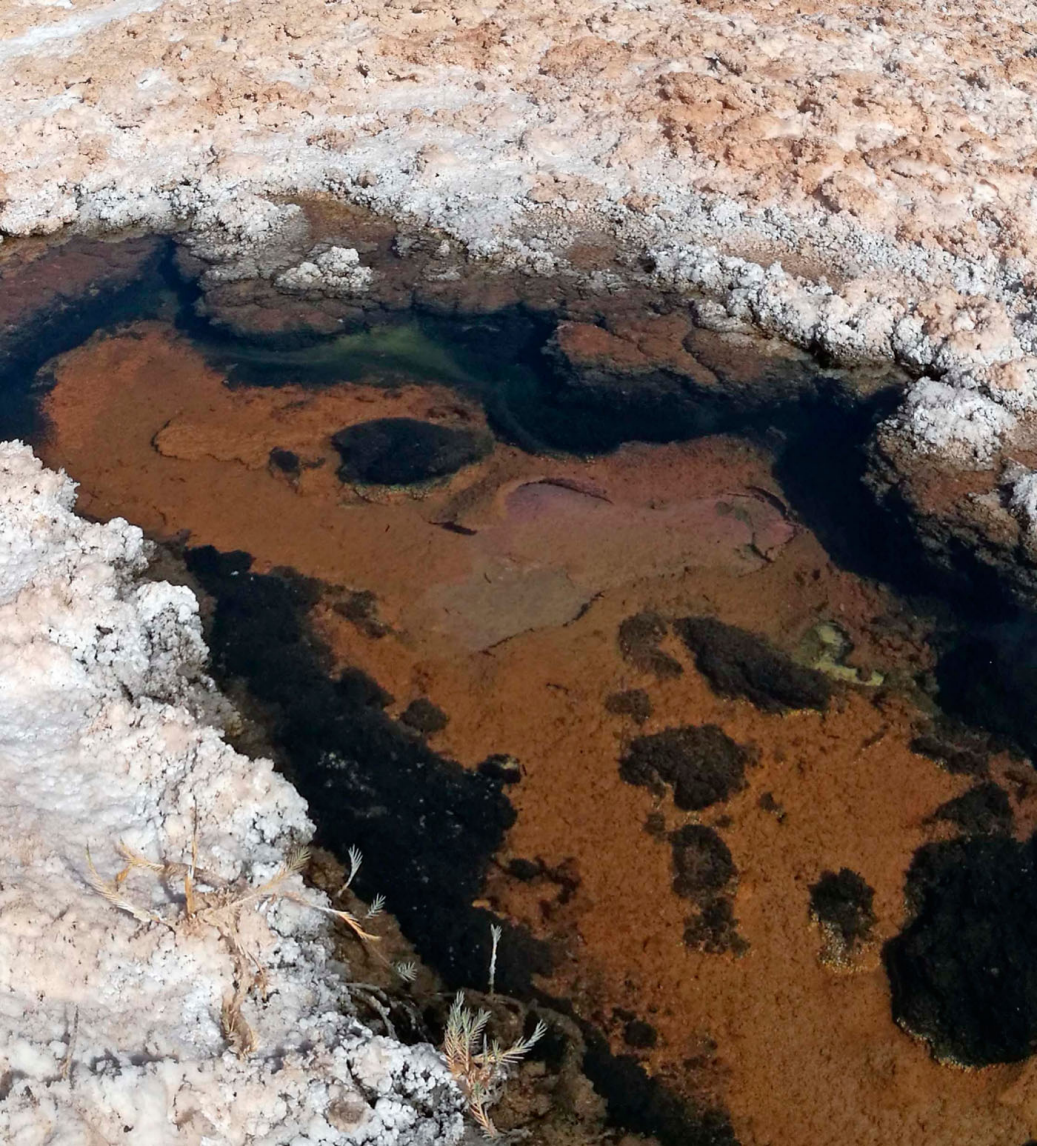
Microbial mat floating on the waters (credit: Cristóbal Bonelli).

The intensity of our outrage, however, only peaked when we found that part of the microbial mat, one of the more visible and colorful forms of microbial life, was floating on the waters (Figure 9).

This sight alarmed and devastated the whole team. For eyes trained by microbiological research on *salares*, the detached piece of matting was the death rattle of an entire community of microorganisms, brought on by the injection of fresh water into a salt water ecosystem containing bacteria over 2500 million years old (Gutiérrez-Preciado et al. 2018). After measuring the electrical conductivity of the waters, the team found a significant change in the salinity of these bodies of water. Since microbial life is adapted to high levels of salinity, a sudden change in salinity levels will disrupt the structure of the microbial mats, breaking off cellular and structural relations among these communities.

As the hours passed, and sad, angry conversations continued, the cosmetic changes to the *salar* and the micro-disaster that we were witnessing increasingly began to affect Cristóbal. Trained originally as a pyschologist, and later as an anthropologist, Cristóbal had cultivated an special sensibility related to human events and their historical experiential times. In his clinical practice, he had become accustomed to hearing - and to be affected by-, the biographies of individuals and families with different histories of suffering. Moreover, Cristóbal’s most recent ethnographic research had focused on Chilean territories deeply affected by state violence, and he had made a considerable effort in trying to conceptualize and to account for the *present of the absent*, of how some absent presences that appear in ethnographic research – alike to the presences of those who were killed in the place where the memorial of Antofagasta was built – have the capacity to affect ethnographers, and their disciplined knowledge, with their spectral force (Bonelli 2019; Bonelli and Poirot 2020).

The affective experience in *Llamara* unexpectedly awakened Cristóbal’s commitment to thinking about how multiple temporalities distinctly persevere in their existence, complexifying our temporal horizons. Nevertheless, the emotions that the destruction of the microbial ecologies triggered were related to a type of affect that exceeds those affects belonging to human historical times. These affects, rather, were provoked by deep-time *salares* and their microbial ecologies threatened by extractivist activity. Unexpectedly, the outrage felt by the microbiologists, and eventually also by Cristóbal, turned into a type of affect that experientially connected the temporalities of biological evolution with historical human time.

**Figure 10.**
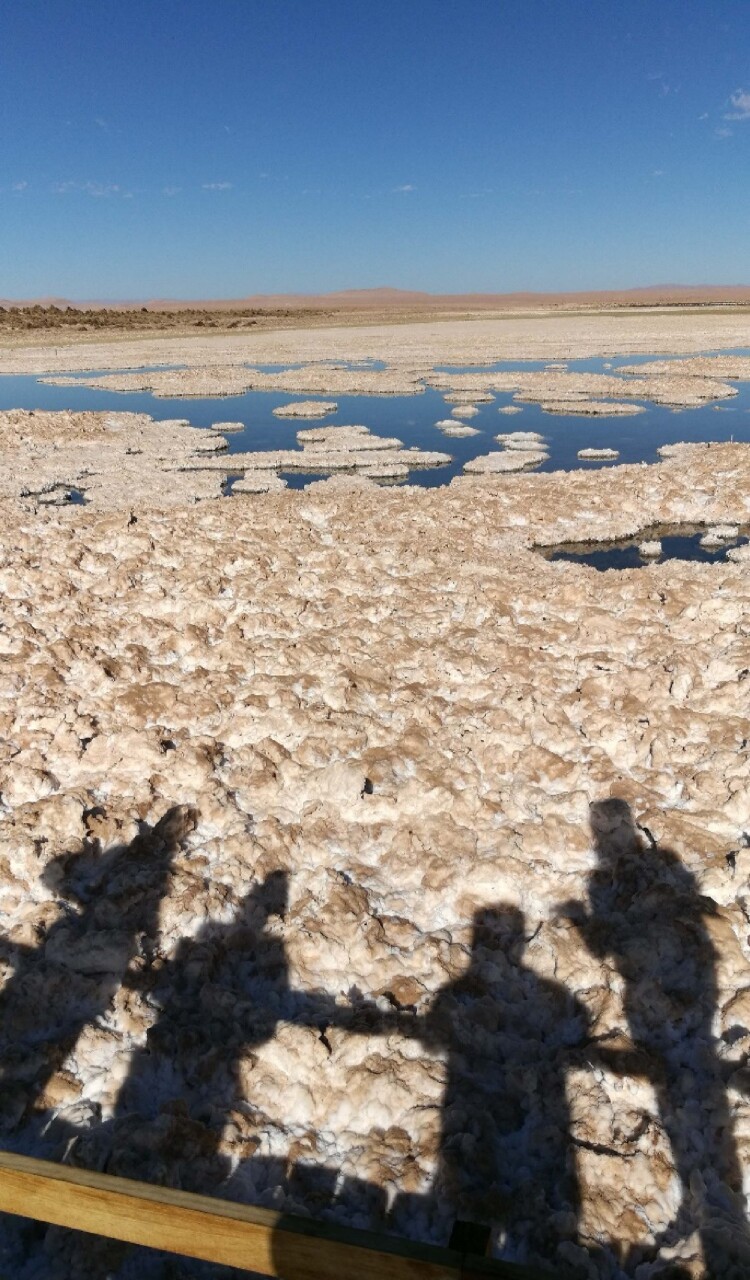
A *therapeutic* conversation in the *salar* (credit: Cristóbal Bonelli).

**Figure 11.**
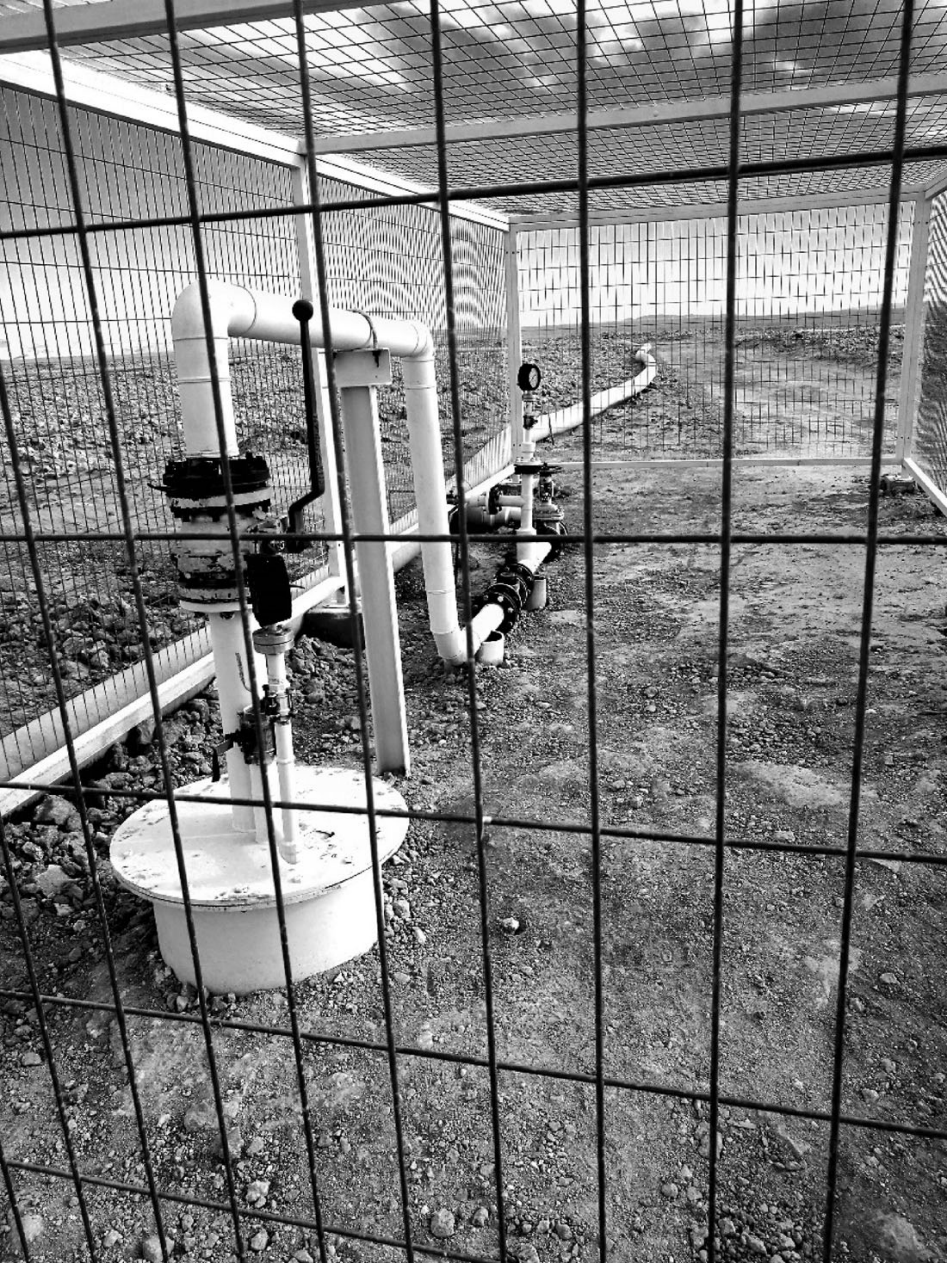
Mining pipelines in the *Salar de Llamara* (credit: Cristóbal Bonelli).

After a couple of hours of conversation, during which the microbiologists shared their frustrations, Cristina joked, “OK, now we have unburdened ourselves, we have done psychotherapy with Cristóbal! Thank you, Cristóbal! All of this is very, very frustrating and we can’t talk about it in our scientific writings, we aren’t trained for that” (Figure 10).^17^ Four years later, nevertheless, we have come to see that this joke expressed in part what we have come to think of as *deep-time affection*, a type of affect that connects historical time with the temporal scales of biological evolutive time – a type of affect, moreover, that puts into question a certain patriarchal naturalism that prohibits emotions from scientific descriptions. Witnessing how the archive of the Great Oxidation Event’s microbial deep-time was disrupted by extractivist activity in fact pointed to the necessity of rethinking not only psychotherapy – bringing biological evolutive time into the everyday! – but also of rethinking ecological reparation from the perspective of planetary processes’ temporalities that exceed human historical time (Figure 11).

Preliminary findings suggest that the waters of the *Salar de Llamara* are extracted, transported about 8 km north of the *salar*, and used to dissolve *caliche* rocks, which produce different types of nitrates used to make solar thermal salts, and eventually, “clean energy,” as these salts, in turn, are put to work for thermal storage and heat transfer in concentrating solar power (CSP) plants.

In fact, the Cerro Dominador CSP plant is beginning operations in the middle of the Atacama Desert. It will be the largest such plant in Latin America, with a capacity of 110 KMW and 17.5 h of thermal storage. Its main tower is 220 meters tall, affording views throughout the desert. The salts it relies on to function are abundant in the Atacama: potassium nitrate and sodium chloride. The potassium salts come from the brines of Atacama *Salar* – an area that also attracts the world’s largest lithium companies – and the sodium nitrate salts (saltpeter) are derived from the plain’s *caliche* deposits using a process that involves water from the *Salar de Llamara* and the Pampa del Tamarugal aquifer.

We do not know exactly what use was made with the water that was extracted, and eventually replaced with fresh water from *Llamara*. What we want to show with the event witnessed in *Llamara* is how *salares* in northern Chile are deeply affected by groundwater extraction, and even by allegedly “small” changes in these water bodies: in the case of *Llamara*, putting the *salar*’s waters to work led to biological annihilation. But what does this annihilation entail for the deep-time microbiological imagination?

## 4. Microbial mats: the ancestral sentinels of the sun

**Figure 12.**
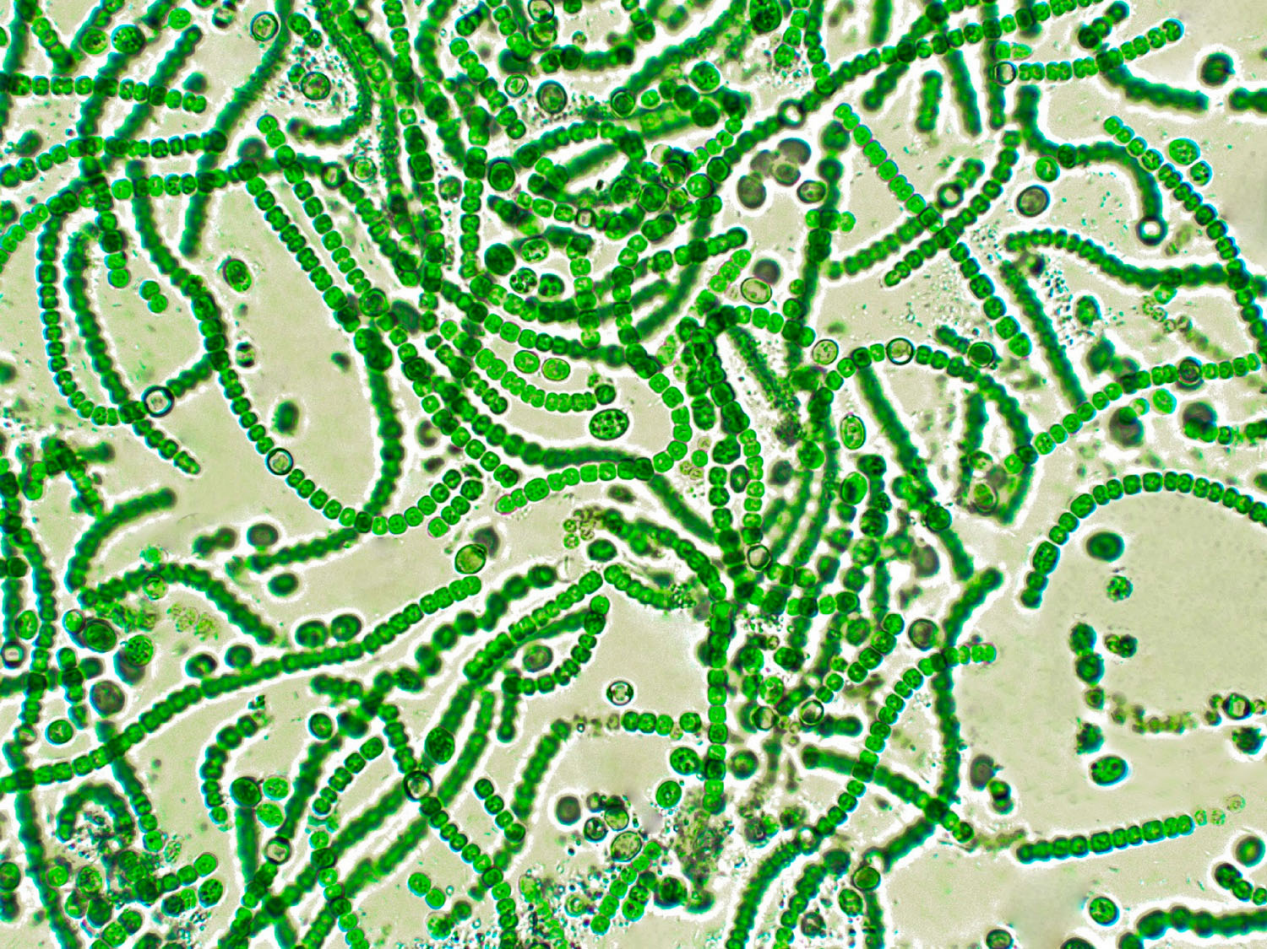
Cyanobacteria under the microscope (credit: Elif Bayraktar/ iStock).

Within micro-biological imaginaries, microorganisms are the first organisms on Earth, and their evolution allowed the oxygenation of the planet 2.5 billion years ago and together caused the biodiversity explosion. As the Earth’s early atmosphere oxygenized, the prevalent physical and chemical conditions were transformed, opening a window of opportunity for change, for diversification. Furthermore, for the biological imagination, life is based on carbon, either as a structural component or as part of cell metabolism (Figure 12).

Let’s have a look at cyanobacteria under the microscope: bacterial cells are formed by different carbon-based molecules (proteins, sugars, lipids, nucleic acids). They are photosynthetic microorganisms, transforming atmospheric carbon dioxide (carbon dioxide fixation) into organic molecules using solar energy. Indeed, in biology decarbonization means to fix carbon dioxide for further purposes, from the atmosphere into the cell. Also, some microorganisms can use carbon monoxide to obtain energy in extreme environments (Cordero et al. 2019). Original microbial cells (microorganisms) used inorganic compounds to obtain energy. They therefore emerged as a quintessential link between the non-organic world and the rising organismal world.

Microorganisms can obtain their energy and carbon from organic and inorganic sources. The most ancient metabolisms were inorganic, existing in intimate connection with their surroundings. It is thought that the ancestor of cellular life on Earth – the so-called Last Universal Common Ancestor – was an anaerobic microorganism that was a hydrogen-dependent, thermophilic fixative of carbon dioxide and nitrogen (Weiss et al. 2016). Life itself began as the complexification of the assemblage with the inorganic. Broadly speaking, microorganisms use and degrade different organic and inorganic compounds because they contain specific enzymes for such functions. Despite their small size and apparent invisibility, they are ubiquitous, diverse and abundant, living in communities and interacting with all other organisms. Microorganisms are the invisible and silent network of the biosphere.

Despite the apparent invisibility of the microorganisms, and although they may need microbiologists to make them visible, they can be visually detected in certain environments. For example, patchy green areas in *Salar de Huasco* are a congregation of microalgae and cyanobacteria. They are green because they contain a pigment called chlorophyll, crucial for photosynthesis.^18^ In oceans, chlorophyll is part of organisms such as cyanobacteria, micro and macro-algae. These organisms are responsible for 40% of carbon fixation on Earth.

**Figure 13.**
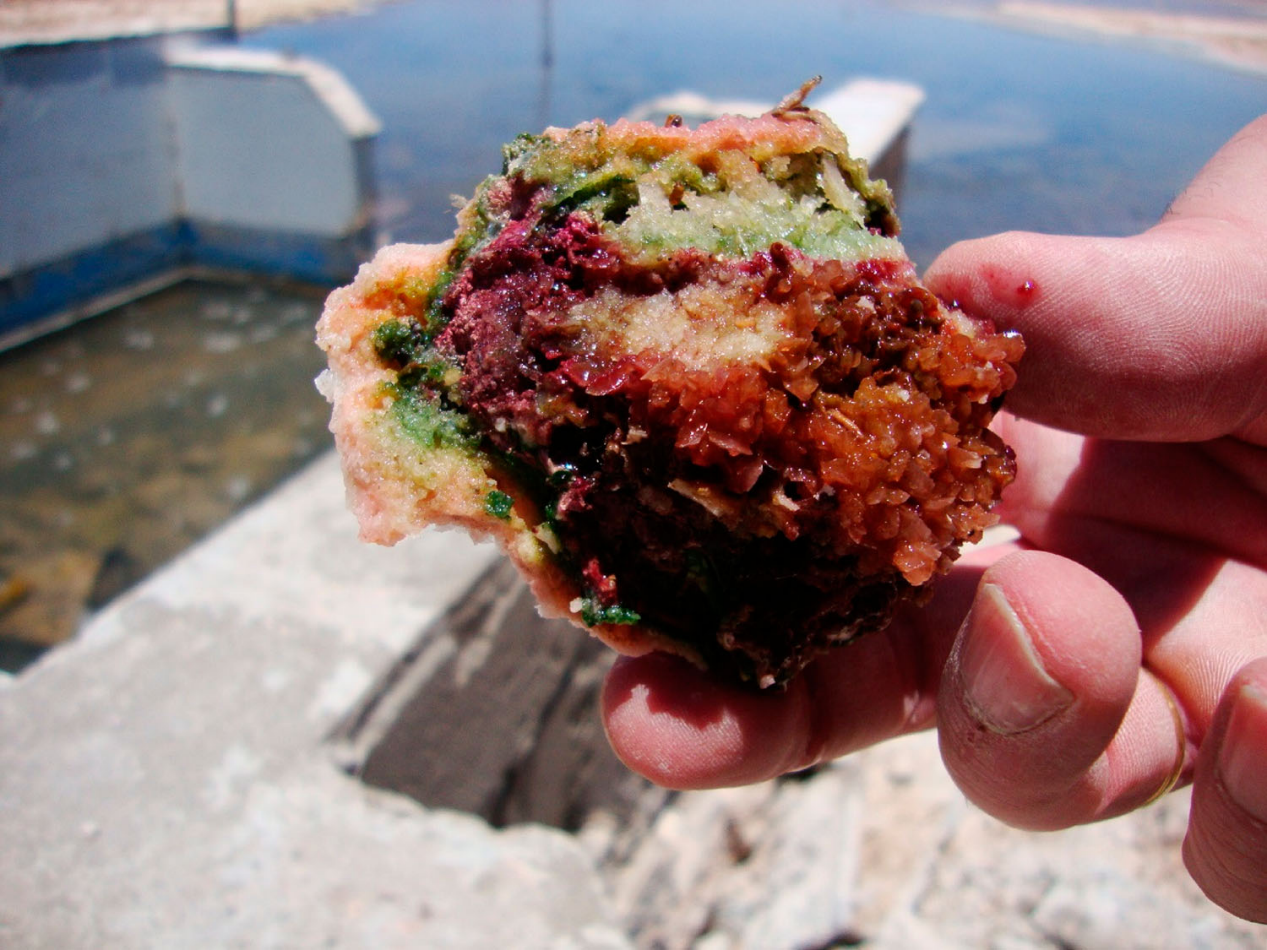
A colorful microbial mat (credit: Cristina Dorador).

Microorganisms interact with carbon on different levels and in different places on the planet. Even viruses, which are not typically recognized as being alive, are considered to be the ocean’s “carbon pump” (Staff 2020), since they release carbon when they infect oceanic microbial cells (phytoplankton, bacteria, and others). Organisms such as zooplankton then consume the released carbon, and either bury it in the sediments of the ocean floor or are eaten by other organisms. Recent studies suggest that the rates of carbon assimilation are far higher than previously thought, as past research had not accounted for various oceanographic factors such as the depth of light penetration (Buesseler et al. 2020). With these understandings about microorganisms in mind, we can better appreciate microbial mats in normal conditions (Figure 13).

Microbial mats are colorful structures located under the extension of the white salt crust of *salares* like *Salar de Llamara*. Each color of the microbial mat indicates a different type of microbial community undergoing a dynamic chemical, genetic and physical intraction. Pink, orange, green, purple, brown and black, each color represents bacterial pigments of different groups: *Roseobacter*, *Sphingomonas*, *Leptolyngbya*, *Chromatium*, *Bacillus*, and *Desulfovibrio*, respectively. Each layer captures different portions of the solar spectrum: a *fair* distribution of solar energy*.* Different wavelengths of solar radiation are detected by different pigments and, consequently, by different microorganisms. The diversity of microorganisms is adapted to the diversity of the solar spectrum. Each photosynthetic community fixes carbon dioxide, converting it into organic matter. Organic molecules are in turn an energy source for other microorganisms. Microorganisms are a food source of zooplankton, insects, and protists. This continuous pipeline of carbon transformation and harmonic development occurs even under extreme conditions.

In conclusion, each microbial community of the microbial mat harbors different microbial populations and species, each with a unique complex singularity in continuity with their environments. They modify the environment and the environment modifies them, thus somehow enacting the etymology of the old French word “environ”: the condition to be encircled or in a circuit (in-viron) and, at the same time, an action, “to turn,” from *virer*. They are a complex form of heterogeneity ontologically indissociable among themselves. In the microbial mats, microorganisms visually exhibit a clear vertical stratification based on the different colored layers; nevertheless, there are invisible connections between the layers mediated by chemical compounds, electrons, viruses, DNA, gases and other organic molecules. On the surface, microorganisms are directly exposed to high solar radiation, where, together with the salts, they form an effective shield that allows for the distinctive utilization of solar radiation by the other bacteria. Microbial mats are the ancestral sentinels of the sun. Under this microbial layer, cyanobacteria perform photosynthesis, producing high concentrations of oxygen that contribute to the functioning of other organisms. Importantly, as we have mentioned above, microbial life is possible because of the presence of water. In dry and arid ecosystems such as *salares*, water is scarce and so is a limiting factor for the development of life. Water *with* salts *is* microbial life. For instance, brines – water with a high concentration of salts and from which lithium is extracted – contain a wide diversity of bacteria, archaea, viruses, and eukaryotic cells (Cubillos et al. 2018). In this context, the microbial mat together with water and the specific environmental conditions of the Atacama Desert form a temporal and spatial dynamic ecosystem, where the modification of one of these factors, as we have seen in the case of *Salar de Llamara*, could trigger the destruction of microbial ecologies, its deep-time intra-actions and loss of microbial biodiversity.

## 5. Towards a holobiontic mode of survival

The events at *Salar de Llamara* show how the deep-time intimacy of microbial ecologies is disrupted by mining activity. They reveal how extractivist practices destroys the deep-time intimacy inherent in *salares* and their microbial ecologies. Importantly, from the perspective of the micro-biological imagination, the *Salar de Llamara* is an archive that contains the memory of past life on Earth. Its microbial mats recapitulate the Great Oxygenation Event (Gutiérrez-Preciado et al. 2018). The collective deep-time microbial memory present in a small puddle in the middle of the Atacama Desert is being erased by the separatist grammars of extractivism, thus obliging us to think further about how to sustain *planetary habitability* rather than depredation of planetary resources (Langmuir and Broecker 2012). We are accordingly convinced that the disappearance of the desert’s microbial habitats should be seen as a *micro-disaster*, a disaster that, while occurring at the microbial level, affects and threatens the vestiges of the planet’s past life, one of the only – also affective! – connections that remains to *our* origins. This *our* includes us – Cristina and Cristóbal as well as you, dear reader, – in a kind of deep-time multispecies crowd alliance. In fact, “they,” micro-organisms, and their intra-actions, also communicate as a “complex we”: consider that within a healthy adult body “the number of bacteria in the body is actually of the same order as the number of human cells” (Sender, Fuchs, and Milo 2016). Our human bodies are communities of different kinds of cells: human, bacterial, archaeal, fungal – cells that in turn relate complexly to each other. Human *being* involves *being* microorganismal, entailing “the fusions and mergers of the planetmates that have preceded us in the microcosm” (Margulis 1998, 12). We are made of bacteria and archaea, as was the first eukaryotic cell. Different species are the expression of such past and present microbial intractions. Aspects of our cerebral functioning – and so of our thinking and generating knowledge about microorganisms – rely on the bacterial generation of neurotransmitters in our intestines. In short, we are human because we are at the same time bacteria and microorganisms. “We” are, indeed, “holobionts,”

symbiotic assemblages … , which are more like knots of diverse intra-active relatings in dynamic complex systems, than like the entities of a biology made up of preexisting bounded units (genes, cells, organisms, etc.) in interactions that can only be conceived as competitive or cooperative. (Haraway 2016, 60)

And as holobionts, we are deep-time *and* deep-history, which implies, as Penny Harvey (2019) has already suggested, to realize that “the mutually constitutive relationship of human and other-than-human worlds is not under human control, even when human intention appears paramount” (Harvey 2019, 188). The mining industry, however, untangles the interwoven, intra-active threads constitutive of the microbial ecologies of the *salares* we are with. The preservation and conservation of the *salares* and their microbial communities therefore require a profound critique of renewable energy production (Jasanoff 2007; Boyer 2019; Cross 2019; Daggett 2019) and allegedly sustainable, one-size-fits-all techno-fixes (Hulme 2014; cf Nadim 2016) that rely on critical minerals such as the lithium contained in the *salares*’ ancient brines.

At the very least, the effect of modern energy production and the mining on the *salares* require us to question sharply energetic logics that operate through practices, grammars and languages of disconnection, which facilitate the slow, invisible advance of environmental destruction (Nixon 2011). Indeed, microbiological understandings of decarbonization (as microbial carbon fixation) are being silenced and threaten by different understandings of decarbonization dominated by the grammars of extractivism and its ideal of “scalability.” Microbial carbon fixation is indeed an ally of an “environmentally complex we” that needs to be made public and visible. In fact, our allies are micro-organisms – Gaia's “innumerable co-authors” (Stengers 2015) – micro-organisms that, despite the destructive practices stemming from a geological period we might call the *extractocene*, will “effectively continue to participate in [Gaia's] regime of existence, that of a living planet” (Stengers 2015, 47). In light of the fact that microorganisms will survive our extinction as human species, we think it is an opportune moment to visibilize the invisible and learn from them, to be affected by them and by their deep-time, which is also, and simultaneously, our own time.

Our goal in this article has been to offer a conceptual experimentation capable of opening up new possibilities for communication and imagination among affected bodies (Despret 2004; Morita and Susuki 2019); indeed, our collaboration has increased our capacity of learning to be affected by deep-time ecologies. Moreover, by offering a story-telling exercise that exhibits how our affective engagements with *salares* have enabled us to develop a planet-centered mode of thinking (Zalasiewicz 2020), we aimed at decentering a humanocentric notion of the political (Deleuze 1968; Latour 1993; Bonelli 2016; de la Cadena 2019) by proposing a radical reconsideration of humanity itself—as Felix Guattari reminds us in the epigraph at the beginning of this article. Doing so, we reckon, does not mean that we understand *salares*, and their deep-time microbial ecologies, as fully withdrawn from human experience (Harman 2018). What we wanted to show, rather, is how our engagements with *salares* increased the awareness of an understanding of humans “as the work of thousands of millions of years of interaction among highly responsive microbes” (Margulis 1998, 4).

Finally, in the context of this special issue concerned with extinction, therefore, we want to conclude by affirming that thinking about extinction entails, by default, being concerned with the question about how to better conceptualize the unit of survival. We are convinced that our concerns with extinction, as well as with justice, cannot any longer be about humans as considered separately from deep-time ecologies and deep-time affection. And this is the reason why we are working in creating urgent experimental alliances, capable of thinking, feeling, and acting upon a holobiontic mode of survival.

## Supplementary Material

Supplemental Material - Spanish Translation

## Footnotes

1 https://www.iea.org/reports/the-role-of-critical-minerals-in-clean-energy-transitions.

2 Minera Escondida has recently announced that it would invest 93 million dollars to repair damages to the *Salar de Punta Negra*, neglecting the fact that “biodiversity recovers only on time-scales of millions of years” (Langmuir and Broecker 2012, 591).

3 Information about this lawsuit is found at: https://www.diarioconstitucional.cl/wp-content/uploads/2020/11/Demanda_Codelco-Salvador__aprobado_CLM.pdf.

4 For the development of this point, see Ampuero (2016).

5 For some paradigmatic examples of the water crisis triggered by this model see Yáñez and Molina (2011); and Bauer (2015).

6 See Weinberg (2021) for recent debates over mineral extractivism in Chile, and the relationship between bodies, work, and mining in the context of neoliberal Chile.

7 Chile is the world’s primary producer of copper, yielding 5.5 million tons in 2016. Escondida mine, owned by a group of private corporations, is the largest copper mine in the world. (Pietrzyk and Tora 2018).

8 We are fully aware that microorganisms have also been exploited by capitalistic logics (see Labban 2014). However, in this article we want to think about microbial dynamics that might endure beyond capitalist hegemonies. We thank Sebastian Ureta for having pointed this out.

9 For an interesting reflection on scalability and lithium, see Köppel 2020.

10 The consideration of these simultaneous temporal scales expands analytics of movement among scales in times of environmental destruction (Hetch 2018) thus transforming humanocentric understandings of scales themselves.

11 This point resonates with Westermann’s examination of how terrestrial and geopolitical scales mix in the Atacama desert (Westermann 2020).

12 Developments in microbiology resulting from collaborations with physicists and chemists have, since 2010, led to a series of “next generation sequencing” (NGS) techniques. NGS allows scientists to analyze DNA from large-scale environmental samples without the need for cloning, thereby eliminating the costs and time associated with cloning.

13 Microbiologists designate species specificity using operational taxonomic units (OTUs), and more recently, in the era of mass sequencing, amplicon sequence variants (ASVs).

14 For Helmreich, infranature is a concept that “keeps analytically audible the continued durability of the concept of nature, especially as it may exist for many scientists, engineers, and architects,” (2016, 84).

15 Etymologically, sympoiesis means “making-with” (Haraway 2016, 58). Haraway mobilized the term to account for how no living being is truly auto-poetic or self-organizing: “Critters interpenetrate one another, loop around and through one another, eat each other, get indigestion, and partially digest and partially assimilate one another, and thereby establish sympoetic arrangements that are otherwise known as cells, organisms, and ecological assemblages” (58).

16 For an account of how Environmental Impact Assessments tend to normalize the current situation and erase environmental memory and devastation in Chile, see Barandiarán (2020).

17 See Law (2015) on how scientific writing obscures the human writer as active agent attempting to discuss nature “out there.”

18 For an inspiring anthropological account of photosynthesis, see Myers (2016).
