# Supplementary material for: Endangered Salares: micro-disasters in Northern Chile
Source: Tapuya. 2021 Oct 7;4(1):1968634. doi: 10.1080/25729861.2021.1968634 (PMC8887917; doi:10.1080/25729861.2021.1968634)
Supplement: Supplemental Material - Spanish Translation [file TTAP_A_1968634_SM9568.pdf]

Salares en peligro de extinción: Micro-desastres en el Norte de Chile[1]

Cristóbal Bonelli[2] y Cristina Dorador[3]

Resumen

Este artículo es fruto de la colaboración transdisciplinaria entre una microbióloga y un antropólogo fuertemente preocupados por la protección de salares del norte de Chile que actualmente se encuentran en *peligro de extinción*. Nuestro objetivo es proponer el concepto de "micro-desastre" como herramienta que examina los modos en que el extractivismo está alterando a estos salares y a sus ecologías microbianas de tiempo-profundo. Estas ecologías son claves para entender eventos tempranos en la Tierra, en tanto la evolución de ellas hizo posible la oxigenación del planeta hace 2500 millones de años, causando así la explosión de biodiversidad.

Considerando como el *ser humano* implica un *ser micro-organísmico* -y como el tiempo humano está enredado con un tiempo micro-organísmico- este artículo conecta la historia extractivista neoliberal chilena con la historia de la evolución geo-biológica. Por lo tanto, mostramos como estos "micro-desastres" nos afectan fuertemente como humanos complejos, y nos obligan a desarrollar, una y otra vez, un modo de colaborar, pensar, sentir y actuar fuertemente centrado en el planeta. En el contexto de este número especial interesado en repensar la 'extinción', insistimos en la necesidad de considerar los problemas puestos por la posibilidad de extinción humana en continuidad con ecologías de tiempo-profundo. Proponemos repensarnos como humanos como un "nosotros ambientalmente complejo", simultáneamente enredado en el tiempo histórico experiencial y el tiempo microbiano profundo.

Palabras clave: Desierto de Atacama; transdisciplinariedad; salares, ecologías microbianas, extractivismo, tiempo-profundo; sobrevivencia

[1] Esta es una traducción al castellano del texto publicado en revista Tapuya 'Endangered *Salares*: micro-disasters in Northern Chile'. https://doi.org/10.1080/25729861.2021.1968634

[2] Departamento de Antropología, Universidad de Amsterdam, Holanda

[3] Departamento de Biotecnología, Universidad de Antofagasta, Chile

"*Existe al menos el riesgo que no haya nunca más historia humana, a no ser que la humanidad se comprometa a realizar una reconsideración radical de sí misma*"

Félix Guattari, Las Tres Ecologías (2000, p.68)

Este artículo es fruto de una colaboración en curso entre los autores de este texto. Nos conocimos trabajando en el norte de Chile, nuestro país de origen, en un lugar que ha sido fuertemente marcado por el extractivismo minero, el desierto de Atacama. Desde nuestro primer encuentro en Atacama, hemos estado cultivando una amistad y aprendiendo mutuamente de nuestras trayectorias y sensibilidades disciplinarias. El desierto de Atacama es un lugar dominado por el extractivismo, a saber, por la 'apropiación de los recursos naturales en grandes volúmenes y/o a gran intensidad, y en donde la mitad, o más de esos recursos naturales, son exportados como materia prima, sin procesamiento industrial, o con limitado procesamiento industrial' (Gudynas 2018, 62). Desde este lugar, nuestro actuar ha estado siempre motivado por el deseo de proteger diversos ecosistemas y formas de vida amenazados por las industrias extractivas; de hecho, este artículo emerge desde nuestra necesidad compartida por narrar -y dar forma- a las maneras en que las transformaciones gatilladas por el proyecto extractivista nos han afectado, y nos siguen afectando hasta el día de hoy. Además, en este trabajo nos interesa considerar cómo nuestra colaboración transdiciplinaria, y nuestros modos de relacionarnos con los hábitats microbianos de tiempo-profundo del desierto, han aumentado nuestra capacidad para actuar, pensar y sentir (Deleuze 1968).

Cristina es microbióloga, y Cristóbal es psicólogo y antropólogo. Desde que nos conocimos por primera vez, en Marzo de 2017, hemos estado experimentando con crear una alianza que tenga 'el poder de hacer pensar y actuar juntamente a las personas preocupadas o interesadas en determinado asunto, haciendo así posible que cada uno de los involucrados se conecte con las formas en las que otros llegaron a estar preocupados o interesados en determinado asunto' (Stengers 2018, 93, *nuestra traducción*). A través de nuestras conversaciones, correspondencias, y visitas a los salares, hemos afiatado nuestro deseo de realizar un experimento transdiciplinario que permita articular nuevas historias ecológicas y maneras de

pensar, que a su vez, sean capaces de intensificar el modo en los que otras personas puedan también sentirse preocupadas con nuestras preocupaciones.

Nuestro mutuo aprendizaje ha expandido nuestro modo de ser conscientes de la profunda historia geo-biológica de los salares; en este proceso, y quizás sin proponérnoslo, hemos también cultivado lo que preliminarmente llamamos un *afecto-de-tiempo-profundo,* un tipo de afecto que, en nuestro caso, se ha ido enmarañando con las maneras en las que la historia neoliberal de Chile también ha afectado nuestras formas de ser, pensar, y sentir. En este sentido, e inspirados en el historiador postcolonial Dipesh Chakrabarty (2021), este artículo conecta dos temporalidades que nos han afectado *simultáneamente*: una temporalidad humana, que en particular refiere a la temporalidad de la historia reciente del Chile neoliberal, y una temporalidad de tiempo-profundo, propia de la evolución biológica. De hecho, siguiendo a Chakrabarty, consideramos esencial considerar seriamente la temporalidad de la evolución biológica en tiempos del Antropoceno: 'Si nosotros no consideramos la historia de los procesos de la Tierra, los cuáles *sacan de escala* (inglés, *out-scale*) nuestros modos muy humanos de comprender el sentido del tiempo, no lograremos concebir, realmente, la profundidad de los dilemas y problemas que como humanos estamos enfrentando en el día de hoy' (156*, énfasis y traducción nuestra*).

Los *salares* son tiempo-profundo: ellos son vestigios de antiguos paleolagos que se ubicaron en la actual zona norte de Chile, norte de Argentina y sur de Bolivia. Debido a distintos procesos geológicos y tectónicos, actualmente son cuencas evaporíticas cerradas que se ubican en la zona altiplánica de los Andes. Al haber sido lagos en el pasado, los salares aún contienen agua de forma subterránea o de forma superficial. Las aguas subterráneas de los salares ubicados en la zona hiper-árida del Desierto de Atacama, tienen una alta concentración de nitratos ('caliche'), boro, y litio, y han sido también minados para extraer el salitre desde el final del siglo 19. En comparación con la historia de tiempo-profundo de los salares, que incluye los periodos de su formación geológica y su evolución biológica, la historia de la economía neoliberal extractivista en Chile, su tiempo histórico, es extremadamente insignificante. A pesar de estas temporalidades desproporcionadas, la historia del extractivismo ha causado daños irreparables. En décadas recientes las compañías mineras han explotado exitosamente las aguas subterráneas y salmueras de los salares, explotación que ellas mismas tienden a justificar apelando a la necesidad actual de implementar transiciones energéticas a nivel global: los salares sacian la sed de litio para baterías y sales de fusión para el almacenamiento de energía solar. Actualmente, esta tendencia extractiva está en expansión.

De acuerdo a un reporte reciente publicado por la Agencia Internacional de Energía, existe un desajuste amenazante entre las ambiciones climáticas globales y la disponibilidad de 'minerales críticos' considerados esenciales para realizar estas ambiciones. Por ejemplo, se prevé que la demanda de litio crecerá 42 veces en el caso que se alcance el objetivo de 'cero emisiones' antes del año 2040.[4] Considerando que más de la mitad de los depósitos de litio del planeta se encuentran en los ambientes áridos y de altas altitudes de los salares Sudamericanos, este crecimiento de la demanda podría ser devastador en términos ambientales para la región. Así, transiciones energéticas que dependen de minerales críticos son -para decirlo suavemente- inconsistentes si no incluyen una crítica paralela del extractivismo y sus impactos ambientales (Babidge 2018; Jerez, Garcés, and Torres 2021; Agusdinata et al. 2018; Liu, Agusdinata, and Myint 2019). Además de esto último, en este artículo sugeriremos que estas transiciones son altamente inconsistentes si ellas no consideran los tiempos-profundos planetarios de las ecologías microbianas.

En Chile, los salares han sido fundamentales en posibilitar el desarrollo económico desde mediados y finales del siglo XIX hasta la actualidad, un desarrollo económico extractivista basado fundamentalmente en 'la extracción acelerada de recursos naturales que satisfacen la demanda global por minerales y energía, proveyendo lo que gobiernos nacionales consideran como crecimiento económico' (Blaser and de la Cadena, 2018, 2; ver Gudynas 2010; Bebbington, 2015; Li 2015; Svampa, 2015). Este proyecto globalizante, con su éxito económico asociado a profundas desigualdades de distribución, ha tenido altos costos y ha dañado en modo permanente a ecosistemas únicos constituidos de relaciones de interdependencia de tiempo-profundo irrepetibles. Por ejemplo, el Salar de Lagunillas (Región de Tarapacá, 3800 msnm) tiene un daño ambiental irreparable al habérsele extraído aguas por más de 20 años para la producción de cobre. Misma suerte corrió el Salar de Punta Negra (Región de Antofagasta, 2945 msnm), donde el Consejo de Defensa del Estado acusó en abril de 2020 a Minera Escondida/BHP de ocasionar un 'daño ambiental continuo, acumulativo, permanente e irreparable' debido a la extracción de aguas desde comienzos de los años 90. Recientemente el mismo Consejo de Defensa del Estado presentó una demanda contra la Corporación Nacional del Cobre de Chile (CODELCO), entidad estatal y una de las principales productoras de cobre a nivel mundial, por el daño "continuo, acumulativo, permanente e irreparable" que sufrió el Salar de Pedernales luego de 36 años de extracción de

[4] https://www.iea.org/reports/the-role-of-critical-minerals-in-clean-energy-transitions

agua[5] . Incluso, en febrero de 2020 se autorizó un nuevo proyecto minero en la zona y se otorgaron otros 47 años de permiso para explorar el agua del mismo Salar de Pedernales. Bajo esta misma lógica productivista, otros salares siguen siendo pensados, enactuados y explotados como recurso, como fuente de agua para uso industrial o directamente como fuente de sales. Las aguas del Salar de Llamara y de la Pampa del Tamarugal, que inspiran en gran parte la escritura de este trabajo, son usadas para establecer pilas de evaporación para la obtención de sales de nitrato y potasio. Las aguas del acuífero de la Pampa del Tamarugal están sobreexplotadas y su uso se ha incrementado en 1890% en los últimos 30 años (Viguier et al. 2019).

Nuestras expediciones conjuntas a los salares del norte de Chile comenzaron en 2017. Desde entonces, hemos estado escribiendo este artículo a medida que íbamos compartiendo nuestras preocupaciones durante largas conversaciones en Santiago, Amsterdam, San Pedro de Atacama, y Antofagasta, la ciudad donde uno de nosotros (Cristina) vive, ciudad que creció durante la edad de oro del salitre y es, hasta el día de hoy, una ciudad marcada profundamente por el extractivismo minero. Durante estos años, nuestros modos de involucrarnos afectivamente con los salares, han ayudado a que nuestra conciencia de tiempo-profundo se expanda, y que al mismo tiempo, nutra los afectos de nuestra propia colaboración. Además, nuestra experiencia histórica compartida de haber crecido en Chile en tiempos de dictadura -y haber sido testigos de la implementación de su proyecto neoliberal-, se ha entrelazado con el tiempo-profundo de la evolución biológica y sus escalas de tiempo (Davies, 2016). En este artículo quisiéramos mostrar cómo tuvo lugar este entrelazamiento de temporalidades.

1. El Globo y su mal-estar

En estos años de colaboración e investigación conjunta, nunca olvidaremos un momento que nos reveló, en una imagen muy nítida, todo el trabajo, violencia, y destrucción que necesitó el proyecto extractivista neoliberal chileno, para ser implementado dentro una expansiva escala global. Esta imagen nos muestra cómo la implementación del proyecto de globalización acelerado, con su propio tiempo histórico, en Chile implicó la imposición violenta de políticas neoliberales bajo la dictadura de Augusto Pinochet (1973-1990).

[5] Para más información al respecto, ver https://www.diarioconstitucional.cl/wp-content/uploads/2020/11/Demanda_
Codelco-Salvador__aprobado_CLM.pdf

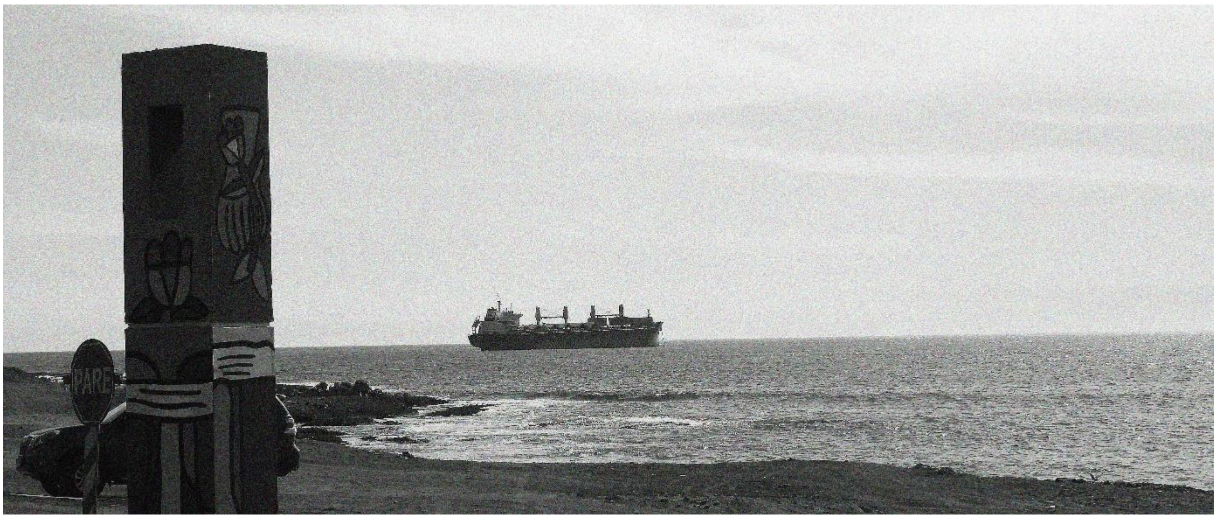

(Figura 1). Barco con minerales alejándose de la costa de Antofagasta, observado desde el memorial dedicado a las víctimas de la dictadura chilena ejecutadas por la 'caravana de la muerte' en Antofagasta (foto: Cristóbal Bonelli)

Tomamos esta foto en una tarde del 2018, después de haber visitado Coloso, un puerto al sur de Antofagasta desde donde inmensas cantidades de concentrado de cobre son drenadas antes de ser exportadas. Cuando íbamos de vuelta a Antofagasta, hicimos una parada en el memorial dedicado a las víctimas de la dictadura chilena ejecutadas por la 'Caravana de la muerte', una comitiva armada del Ejército de Chile que sobrevoló Chile de sur a norte entre el 30 de Septiembre y el 22 de Octubre de 1973.

La foto muestra uno de los pilares del memorial ubicado frente al mar; en el mar, podemos observar un barco alejándose de la costa. Ubicado entre las montañas y el mar, el memorial preserva el dolor del asesinato de 14 personas ejecutadas por agentes militares de la dictadura el día 19 de Octubre de 1973, a la 1.30 de la madrugada. Estas 14 personas asesinadas en Antofagasta fueron ejecutadas con sus ojos vendados; no se les permitió ni siquiera mirar las estrellas por una última vez. Compartimos un largo momento de silencio en este lugar, ambos sintiéndonos profundamente afectados por la historia de la violencia política chilena, y por su tiempo histórico.

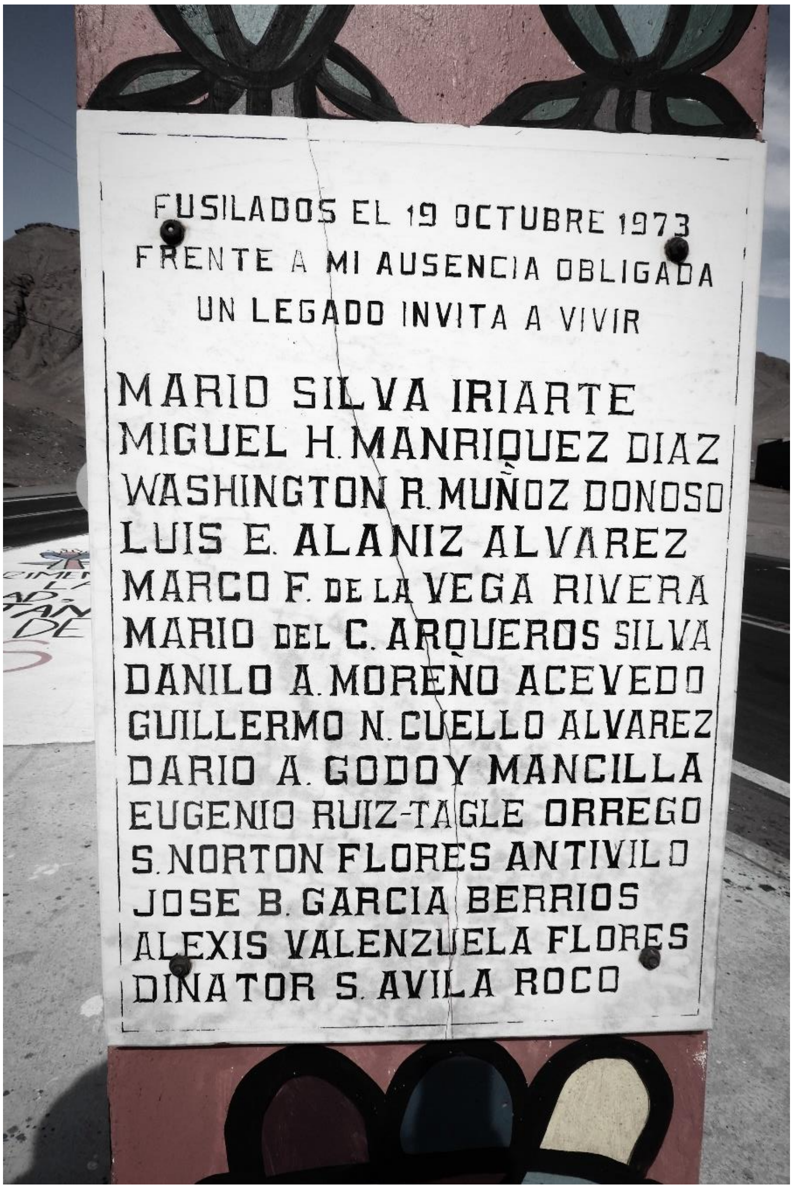

(Figura 2). Nombres de las 14 personas asesinadas por la Caravana de la muerte el 19 de Octubre de 1973 (Foto: Cristobal Bonelli)

En el interior del barco, se almacena una mezcla oscura de minerales y agua: seguramente el barco lleva concentrado de cobre, aunque también, barcos como este, podrían llevar carbonato de litio, o en tiempos pasados, simplemente salitre. El barco viaja por el océano con rumbo a fundiciones e industrias en otras latitudes, probablemente China, a juzgar por nombre del barco escrito en mandarín.

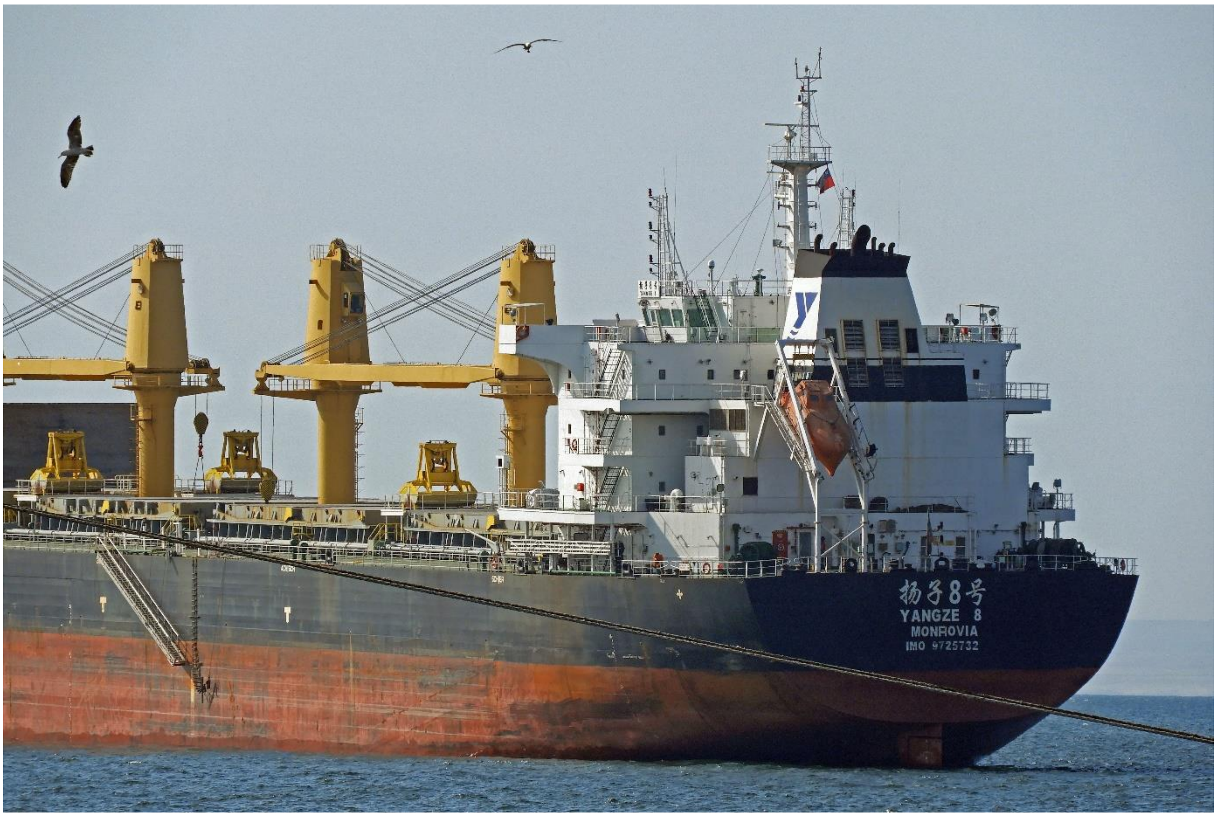

(Figura 3). *Close up* al barco con nombre en mandarín (Foto: Cristóbal Bonelli).

Aunque no tenemos espacio en este trabajo para desarrollar un recuento histórico de las relaciones económicas entre China y Chile, vale la pena mencionar que durante los años 80, la Republica China -en concordancia con sus reformas económicas del 1978-, reactivó relaciones comerciales con la dictadura chilena. Los líderes chinos estaban particularmente interesados en el proyecto económico Chileno, que ellos consideraron como una exitosa liberalización de la economía sin democratización política.[6] Por ejemplo, la dictadura militar privatizó las aguas chilenas, transformándose en un líder internacional en políticas de agua pro-mercado. El código de agua de 1981 introdujo una separación radical entre la tierra y el agua, definiendo al agua como propiedad privada sin regulación estatal significativa. Este código ha gatillado problemas de desigualdad, conflictos entre usuarios de agua, y un crisis ambiental severa.[7]

[6] Para el desarrollo de este punto, ver Ampuero (2016).

[7] Para conocer ejemplos paradigmáticos de esta crisis hídrica gatillada por este modelo, ver Yáñez and Molina 2011; Bauer 2015.

Estas fotos aun nos conmueven y recuerdan el momento en que en un modo fuertemente visual, pudimos señalar, constatar y experienciar dolorosamente en nuestros cuerpos, como fue necesario establecer un régimen de violencia institucionalizada para facilitar el extractivismo neoliberal en la historia del Chile contemporáneo.[8] La foto tomada desde el memorial revela la conexión histórica y material que existe entre la matanza de la dictadura -y su manejo brutal de la población- y la instauración del proyecto neoliberal extractivista capitalista, que desde 1973, ha extraído millones de toneladas de cobre transportadas en barcos como este, produciendo así millones de millones de dólares acumulados por capitales internacionales y parte de la elite nacional.[9]

Las fotos nos sugieren en manera vívida como la industria minera es extractivista en un doble sentido: al extraer minerales y al extraer plusvalía en el proceso de trabajo (Labban 2014; Weinberg 2021). En este artículo mostraremos como esta extracción, además, altera la vida relacional de tiempo-profundo presente en las ecologías microbianas de los salares y la historia evolutiva biológica de ellas, historia que intentaremos demostrar, es también nuestra propia historia de evolución biológica.[10] De hecho, en este trabajo nos interesa mostrar cómo, en el norte de Chile, el globo de este proyecto globalizante y el planeta, con sus procesos bio-geo-químicos ancestrales, están conectados: "Lo que los conecta son los fenómenos del capitalismo moderno… y la tecnología, ambas globales en sus alcances" (Chakrabarty 2021, 4). De hecho, estas fotos nos permiten visualizar como, cuando se implementa el proyecto extractivista expansivo, tecnológico, de cadenas globales humano-céntricas que dependen de minerales chilenos, el capitalismo extractivista separa violentamente a organismos de sus ambientes (Bateson 1972). De hecho, el barco y sus caligramas chinos muestran como este proyecto se ha expandido a través de un modo expansivo y desarraigado de producción

[8] Para debates recientes sobre extrativismo minero en Chile, y su relación con cuerpos, trabajo y minería en el contexto neoliberal chileno, ver Weinberg (2021)

[9] Chile es el primer productor de cobre a nivel mundial, exportando 5.5 millones de toneladas en 2016. La mina La Escondida, cuyos propietarios son un grupo de corporaciones privadas, es la mina de cobre más grande del mundo (Pietrzyk and Tora 2018).

[10] Si bien hay estudios que muestran como los microrganismos han sido sujeto de explotación capitalista (Labban 2014), en este trabajo nos interesa pensar dinámicas microbianas que persistirían más allá del capital. Agradecemos a Sebastian Ureta por haber notado este punto.

extractivista, que podríamos pensar, siguiendo a Tsing (2016), como enactuando el ideal de la 'escalabilidad' (ingles, *scalability*).[11] Para Tsing, la escalabilidad es:

> La habilidad de un proyecto para cambiar escalas con facilidad sin realizar ningún cambio en la estructura del proyecto. Un negocio escalable, por ejemplo, no cambia su organización a medida que se expande. Esto es posible solamente si las relaciones de negocio no son transformativas, ni cambian el negocio a medida que nuevas relaciones se van agregando (…) la escalabilidad hace desvanecer relaciones de diversidad significativa, esto es, diversidad que podría llegar a cambiar las cosas (38, nuestra traducción)

Inspirados en estas ideas, y profundamente preocupados por la actual destrucción ambiental en el norte de Chile provocada por la industria minera, en este trabajo queremos mostrar como la escalabilidad de este proyecto extractivista neoliberal expansivo está destruyendo salares y al mismo tiempo haciendo desvanecer la diversidad microbiológica significativa y sus relaciones de interdependencia. En particular, queremos mostrar como, siguiendo el trabajo de Shiho Satsuka (2015), esta escalabilidad ha implicado la transformación de un proyecto de hacer-mundo a otro proyecto de hacer-mundo. De hecho, mostraremos como las relaciones de las ecologías microbianas no son escalables. Sin embargo, al hacer esto, quisiéramos también expandir, de alguna manera, la relación entre este modo de comprender la escalabilidad y el globo del proyecto globalizante y extractivista. Creemos que la definición de escalabilidad como 'la habilidad de un proyecto para cambiar escalas fácilmente sin realizar ningún cambio en su propia estructura' no es suficiente para hacer sentido de las temporalidades propias de los salares y sus ecologías; de hecho, pensamos que puede ser beneficioso, analíticamente, explicitar como la escalabilidad hace visible, siguiendo a Chakrabarty (2021), el planeta:

> Cuando pensamos históricamente sobre los humanos en una época en la que la intensa globalización capitalista ha generado la amenaza del calentamiento global y de la extinción en masa, necesitamos traer a la mano conjuntamente categorías que hemos usualmente usado, en el pasado, como separadas y virtualmente desconectadas. Necesitamos conectar historias profundas e historias escritas, y poner el tiempo biológico y geológico de la evolución en conversación con el tiempo de la historia y experiencia humana (7-8, nuestra traducción)

[11] Para una interesante reflexión sobre escalabilidad y litio, ver Köppel 2020.

Por lo tanto, cuando consideramos procesos planetarios, la escalabilidad no solo refiere a 'la habilidad de un proyecto de cambiar escalas fácilmente sin realizar ningún cambio en su estructura', sino que también implica la conexión entre temporalidades humanas y procesos planetarios bio-geológicos. De esta manera, la escalabilidad relaciona diferentes escalas temporales, 'dos tipos diferentes de 'tiempos-ahora', en manera simultánea' (Chakrabarty 2021; Davies 2016).[12] Al hacer esta conexión, nos parece plausible sugerir que la escalabilidad tiene también el potencial no solo de descentrar a lo humano, sino que también ofrece una oportunidad para llevar a cabo una *reconsideración radical* de lo humano, como propone Felix Guattari en el epígrafe que abre este artículo. De hecho, en este trabajo intentaremos mostrar como la escalabilidad, en cuanto altera el tiempo-profundo de las ecologías planetarias, altera, simultáneamente, nuestra propia coexistencia y continuidad con los salares y sus (nuestras) ecologías microbianas de tiempo-profundo. [13]

En este sentido, esperamos que nuestro trabajo pueda contribuir en hacer visible aquellos micro-desastres invisibles gatillados por proyectos extractivistas. Con el término 'micro-desastre' quisiéramos dar cuenta de un tipo particular de 'desastre lento' (Knowles 2014) en el que el tiempo humano y el tiempo planetario están conectados, un desastre en el que hábitats y relaciones de tiempo-profundo se tornan vulnerables y desaparecen. Profundamente convencidos que la sobrevivencia humana depende en reconocer las relaciones de interdependencia (o intradependencia, como veremos) de tiempo-profundo, en este trabajo nos interesa mostrar como estos micro-desastres ocurren a través de 'relaciones políticas muy densas, en muchas escalas, entre muchos seres vivos entrelazados, no solo microbianos' (Helmreich 2014). Para poder mostrar este punto, es necesario primero examinar brevemente las prácticas microbiológicas que se han venido desarrollado en los salares del desierto de Atacama durante las últimas décadas. Son estas prácticas las que han hecho visible la vida en lugares donde se pensaba, al menos desde la perspectiva de la ciencia moderna, que la vida estaba ausente.

[12] La consideración de estas escalas temporales simultaneas expande analíticas preocupadas por el movimiento entre escalas en tiempos de destrucción ambiental (Hetch 2018), y de esta manera, transforma comprensiones humano-céntricas de las mismas escalas.

[13] Este punto resuena con el trabajo de Westermann y su análisis sobre como escalas terrestres y geopolíticas se mezclan en el desierto de Atacama (Westermann 2020).

2. Microbiología de salares en Atacama

Para la imaginación microbiológica, los microorganismos son los organismos más abundantes y diversos en el planeta. Ellos se encuentran en casi todos los ambientes de la Tierra, incluidos aquellos denominados extremos, que podrían ser considerados como lugares supuestamente hostiles para la vida. La alta diversidad microbiana del planeta se basa fuertemente en la capacidad de dispersión, adaptación y de conexión que generan los microorganismos entre sí y con el resto de las especies. Es decir, el planeta es una red microbiana de interacciones donde los microorganismos son, y continúan siendo, no solo la base y el origen de la vida en la Tierra, sino también parte constitutiva de nuestro *ser humano*, con nuestra historia evolutiva de tiempo-profundo.

Desde una perspectiva microbiológica, por mucho tiempo el desierto de Atacama fue considerado como un lugar sin vida. Sin embargo, gracias a una 'revolución microbiana' gatillada por grandes desarrollos en la secuenciación de ADN, la microbiología ha aprendido que los ecosistemas del desierto están dominados por una alta diversidad de vida microbiana. Estos microorganismos muestran adaptaciones específicas para afrontar una alta radiación solar, desecación, exposición a metales pesados, entre otras condiciones, ofreciendo importantes pistas astrobiológicas para el estudio de 'los limites de la vida' en la Tierra. Además, estas comunidades microbianas han tenido una relevancia esencial en ciclos biogeoquímicos de tiempo-profundo, para al cambio climático, y para la producción de compuestos bioactivos.

Los salares son ecosistemas poliextremos, donde la vida posee una dinámica de bordes. El agua se congela en las noches, pero durante el día hay altas temperaturas y radiación solar. En algunos sectores de los salares se encuentran sales cristalizadas, y en otros, agua dulce. Los salares son lugares donde existe la radiación solar más alta del planeta, donde llega la luz del sol casi sin filtro, y donde los microorganismos están adaptados de alguna manera a esa condición de energía excesiva. Uno de nosotros, Cristina, ha estado estudiando activamente la microbiología de salares del norte de Chile durante los últimos 15 años.

La tesis doctoral de Cristina (Dorador 2007) implicó estudiar muestras que llevó desde los salares del norte de Chile a los laboratorios del Instituto Max Planck en Alemania. Más específicamente, esta investigación doctoral estudió la diversidad funcional y taxonómica de comunidades microbianas de los salares, lagos y humedales del norte de Chile.

Las muestras de salares utilizadas por Cristina permitieron comenzar a conocer la singularidad de los microrganismos, abriendo de este modo la posibilidad de conocer e imaginar una realidad microbiana que se manifiesta en una alta diversificación en cada uno de estos salares. De hecho, hoy, desde la microbiología, se puede afirmar que cada salar en el Altiplano tiene una huella digital microbiana distintiva, y está poblado por comunidades microbianas únicas adaptadas a estos sistemas usualmente conocidos como sistemas extremos, dada las condiciones extremas en la que la vida florece. En términos funcionales y evolutivos, puede ser que los salares se parezcan unos a otros (o sea que sean redundantes funcionalmente), pero este parecido esconde siempre una diversidad microbiana abismante.

Durante estos años de investigación doctoral, las técnicas estándares para conocer y estudiar a los microorganismos correspondían a 'técnicas independientes de cultivo' (basados en el estudio del ADN o ARN de las muestras microbianas). Estos métodos produjeron descripciones de comunidades microbianas secuenciando ADN a través del clonamiento o secuenciación directa de algún gen marcador que diera alguna indicación de diversidad (por ejemplo, el gen 16S rARN).

En el caso de microorganismos de salares estudiados en estos años, trabajar con métodos independientes de cultivo, basados en clonación y secuenciación Sanger, permitió generar una visión limitada de la diversidad microbiana. Más recientemente, la investigación sobre ADN ambiental ha permitido una resolución más alta de la visualización de la diversidad microbiana de salares, sin necesidad de cultivar. Ambientes que se pensaban que no albergaban vida pasaron a ser oasis de vida microbiana. Por ejemplo, fueron visualizadas las

comunidades microbianas que viven dentro de las rocas de halita en el Desierto de Atacama.[14]

Hoy en día podemos obtener millones de secuencias por cada muestra analizada, haciendo de la diversidad una realidad incontable y generando un problema casi ridículo y terriblemente abrumador referido al límite de diversidad en estos salares: *la diversificación es tanta que tiende al infinito*. Es probable también que los distintos algoritmos usados en analizar estas secuencias contribuyan a disminuir este problema de forma técnica. Sin embargo, la pregunta que subyace y permanece abierta es, ¿cuál es la unidad mínima de 'organismo' en un contexto de secuencias de ADN?

Esta bio-lógica del infinito como posibilidad, entonces, puso en jaque no solo a la descripción taxonómica clásica de la microbiología que describe y nombra tipos de bacterias, sino también a la definición misma de especie: se hizo difícil pensar cual podría ser la unidad microbiana mínima de existencia. De hecho, y considerando que la mayoría de los microorganismos no pueden ser cultivados en el laboratorio, se ha vuelto inapropiado asignar especificidad de especie cuando una parte de la secuencia revela una diferencia significativa en relación a otra parte de la secuencia.[15] Es posible que ni siquiera exista la unidad de especie a nivel microbiano, sino simplemente secuencias de diferencias que hacen nuevas diferencias que hacen nuevas diferencias que hacen nuevas diferencias, *ad infinitum* (ver Bateson 1972).

Esta imaginación microbiológica que nos permite pensar la diversidad microbiana *ad infinitum* tiene fuertes implicaciones cuando intentamos asir, o conceptualizar, qué es aquello que se pierde o desaparece en lugares como el desierto de Atacama, lugar fuertemente dominado por las industrias extractivas. Aquello que se pierde, nos parece, son relaciones de tiempo-profundo, relaciones que no son entendidas como productos secundarios de unidades

---

[14] Desarrollos de la microbiología producto de colaboraciones entre la físicos y la químicos, llevaron a una serie de 'técnicas de secuenciación de alto rendimiento' (NGS, *next generation sequencing*). La NGS permitió a científicos analizar el ADN presente en muestras ambientales a gran escala y sin necesidad de clonamiento, lo que redujo significativamente los costos y el tiempo empleado en el clonamiento.

[15] En microbiología se designa la especificidad de especie usando 'unidades operacionales taxonómicas' (OTUs) y más recientemente, en la era de la secuenciación masiva, la secuenciación de amplicones de PCR (en inglés, *amplicon sequence variants*, ASVs).

discretas preexistentes; aquello que se pierde son más bien relaciones entendidas en un continuo e inmanente flujo de relaciones de dependencia e interdependencia.

En nuestras conversaciones transdiciplinarias, este modo de entender las relaciones resonaba fuertemente con el 'realismo agencial' propuesto por Kared Barad (2007), y la problematización que ella hace sobre la usada noción de interacción, ya que esta última supone asumir que entidades independientes preexisten a sus modos de actuar entre ellas:

> En contraste (a la noción de interacción), la noción de 'intracción' desmantela (inglés *queers*) el sentido familiar de causalidad (donde uno o más agentes causales preceden y producen un efecto). En sentido más general, la noción de 'intraccion' desestabiliza la metafísica del individualismo (aquella creencia en la existencia de entidades o agentes -como también tiempos y espacios- constituidos individualmente). Esto quiere decir que 'intracción' tiene que ver con la pregunta por el hacer de diferencias, o de 'individuos', y no con el asumir la independencia y existencia previa de ellos… los 'individuos' solo existen dentro de fenómenos (relaciones particulares materializadas/materializándose) en sus reiteradas reconfiguraciones intractivas en curso. (Barad in Kleinman 2012, 77, *nuestra traducción*).

La manera en cómo la microbiología de salares nos ayuda a comprender estas reconfiguraciones intractivas y al mismo tiempo visualizar cómo las relaciones no preceden a las unidades, ha sido un punto de encuentro fuerte en nuestra colaboración transdisciplinar. Además, la consideración de esta imaginación microbiológica nos ha ayudado a repensar cómo las prácticas extractivistas -entendidas como la ejecución de un modo capitalista caracterizado por el desmantelamiento de 'relaciones de interdependencia' (Stengers 2020a), altera, y a veces, destruye, relaciones de 'intradependencia' de tiempo-profundo en distintos niveles. Esto lo hace a través de la deliberada producción de unidades ambientales separadas, susceptibles a ser extraídas.

Por otro lado, estamos muy conscientes que estos desarrollos recientes en microbiología, al ser examinados a través de sensibilidades propias de los Estudios de Ciencia y Tecnología (CTS), podrían ser considerados como parte de 'infra-naturalezas' (Helmreich 2016) del Desierto de Atacama más vastas, como parte de un desierto 'infra-natural' que ha sido tan arduamente trabajo a través de herramientas científicas, que ha llegado a ser considerado como ocupando el lugar de la 'naturaleza en si misma'.[16] Sin embargo, más que intentar enfatizar ese tipo de análisis, y más que intentar persuadir a una microbióloga que escriba como un antropólogo, o que un antropólogo intente convertirse en un científico de las ciencias naturales, en este artículo queremos experimentar con la mezcla de lenguajes y sensibilidades

[16] Para Helmreich, 'infranaturaleza' es un concepto que 'mantiene analíticamente plausible la durabilidad continuada del concepto de la naturaleza, especialmente en el modo que este existe para muchos científicos, ingenieros, y arquitectos', p 84.

disciplinares, motivados por nuestro compromiso afectivo con los *salares infra-naturales* y sus ecologías microbianas. Este diálogo transdiciplinario entre nosotros revela cómo ecologías microbianas pueden generar un lenguaje de alianza potencialmente capaz de deshacer historias dualísticas que oponen aquello que es humano con aquello que es no-humano (de la Bellacasa 2017). De hecho, y en vez de proponer historias dualísticas, nosotros ofrecemos una manera experimental de reconsiderar lo humano como constituido por relaciones de tiempo-profundo e intracciones que preceden unidades (Haraway 2016). Al proponer esto, estamos inspirados en el trabajo de Margulis y Fester (1991), que muestra como formas biológicas novedosas evolucionan no solo a través de una darwiniana 'descendencia con modificación', sino que también a través de fusiones íntimas y simbióticas de tipos diferentes de células y organismos. El trabajo de Margulis muestra enfáticamente como es imposible conceptualizar entidades únicas aisladas del mundo. Su teoría de la endosimbiosis ha sido clave para entender el origen de los eucariontes; ella demuestra cómo la fusión de una célula bacteriana con una arquea genera un nuevo tipo de célula, la célula eucariota, la cual toma su núcleo de una bacteria (Alphaproteobacteria) y su estructura celular desde un receptor de arquea. El mismo proceso ocurre con células eucariotas vegetales, cuyos núcleos derivan de cianobacterias. Intracciones biológicas y cambios en las condiciones planetarias favorecieron la evolución de nuevas especies, generando el desarrollo masivo de la biodiversidad en el Periodo Cámbrico (la 'explosión Cámbrica'). Y este es el motivo por el que, 'desde la larga visión del tiempo geológico, las simbiosis son como destellos de iluminación evolutiva' (Margulis 1998, 8). Esta teoría ha influido notablemente en el reciente desarrollo del trabajo de la bióloga y feminista Donna Haraway (2016), quien nos ha invitado a desarrollar una 'imaginación simpoietica' para poder sobrevivir en nuestro dañado planeta, una imaginación que nos permita pensar libremente como las unidades no preceden a sus relaciones (Haraway 2016; McFall-Ngai 2017).[17]

En lo que viene, mostraremos como nuestra propia 'imaginación simpoietica' fue nutrida y expandida a través de nuestros compromisos afectivos con los salares y con las maneras en las que hemos sido afectados por el tiempo-profundo de sus ecologías microbianas. Nuestro involucramiento experiencial con los salares ha aumentado nuestra capacidad para sentir las

---

[17] Etimológicamente, simpoesis significa 'hacer-con' (Haraway 2016, 58). Haraway moviliza este término para dar cuenta de cómo no existen seres vivos verdaderamente auto-poeiticos o autoorganizados: 'Las criaturas se interpenetran unas a otras, se dan vueltas alrededor y a través de ellas, se comen entre ellas, tienen indigestión, y parcialmente digieren y parcialmente se asimilan las unas con las otras, y de esta manera establecen arreglos simpoeiticos que llegan a ser conocidos como células, organismos, o ensamblajes ecológicos' (58).

historias de tiempo-profundo de nuestro dañado planeta, cuyos ancestrales procesos geobiológicos están siendo alterados por operaciones extractivistas ‘separatistas’ y por sus gramáticas sujeto-objeto. A continuación, nos concentraremos más detenidamente en un evento particular del que fuimos testigos en Mayo del año 2017.

3. Los afectos profundos del Salar de Llamara

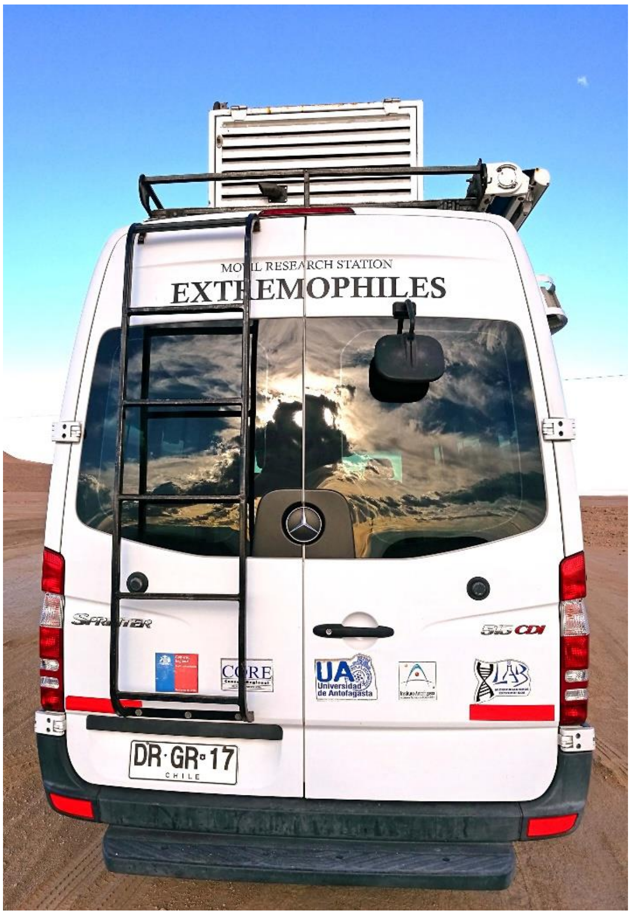

(Figura 4). Laboratorio de investigación móvil sobre extremófilos (foto: Cristóbal Bonelli).

Entre los distintos viajes que hemos realizado a distintos salares desde que nos conocimos, quisiéramos concentrarnos en un evento particular que ocurrió cuando visitamos el Salar de Llamara, un ecosistema salino muy frágil ubicado en el corazón del desierto de Atacama, en la Pampa del Tamarugal, territorio donde también se desarrolló la industria extractivista del salitre a principios del siglo XX. El nombre del lugar proviene del Tamarugo, un árbol nativo del desierto que obtiene agua desde napas subterráneas y que está altamente adaptado a las condiciones ambientales de la zona. Estos árboles tienen en sus raíces unos nódulos con

bacterias que fijan el Nitrógeno, puesto que con suelos tan pobres en nutrientes, no hay otra forma que los tamarugos sobrevivan si no es mediante las simbiosis con bacterias.

La cuenca de la *Pampa del Tamarugal* en el pasado tuvo bosques de tamarugos, los cuales -al ser utilizados como fuente de energía del extractivismo pasado- fueron casi extintos (Castro 2020). La leña y el carbón obtenido del tamarugo se usaba para obtener la energía de la actividad industrial de la plata, el salitre, y era usado como energía para los puertos guaneros de la región de Tarapacá durante los siglos XIX y XX.

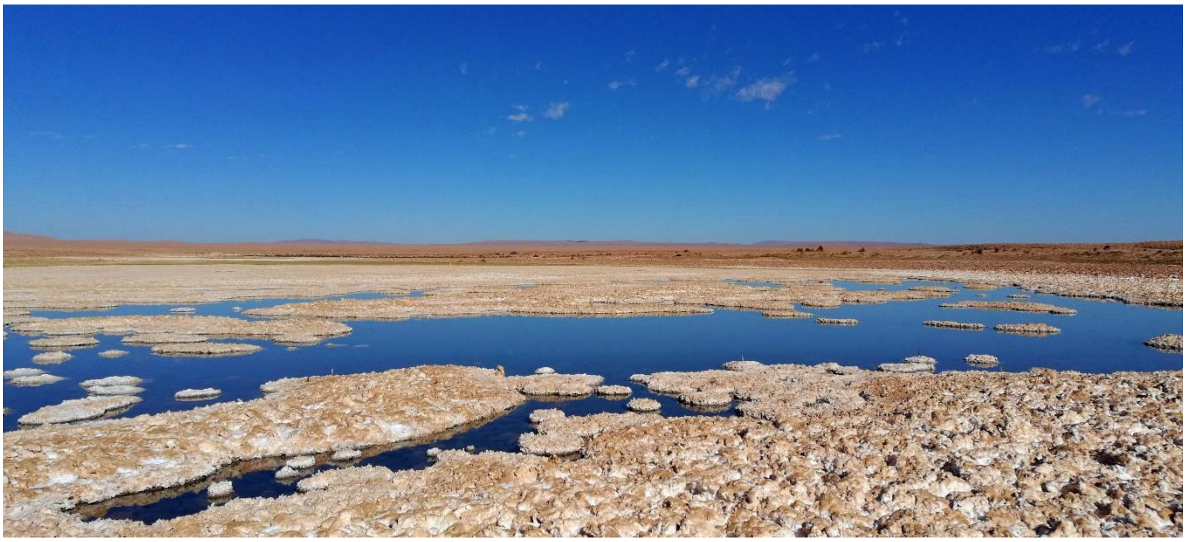

(Figura 5). Salar de Llamara (foto: Cristóbal Bonelli)

Este salar está ubicado dentro de la Reserva Nacional Pampa del Tamarugal (creada el año 1987), y es el único salar con agua superficial de la zona. Llamara ha sido estudiado previamente desde la microbiología por sus formaciones de yeso que forman estructuras similares a estromatolitos (Demergasso et al. 2003). Además, metabolismos microbianos muy antiguos que se han detectado en este salar, como la oxidación de monóxido de carbono (King 2015), han llamado la atención de astrobiólogos; los tapetes microbianos de este salar son considerados claves para entender eventos de la Tierra primitiva, como el gran evento de oxigenación, momento en que las bacterias (específicamente, cianobacterias) comenzaron a producir oxígeno (Gutiérrez-Preciado et al. 2018).

Además, a diferencia de otros estudios abocados a estudiar la evolución de la vida desde su inicio hasta el presente, este salar es único en cuanto permite no solo estudiar la evolución que pudo haber ocurrido en la Tierra, sino también su *la evolución hacia la muerte*. Por eso se dice que Llamara es un sistema terminal, un sistema que está en letargo, y donde se puede estudiar cómo es que la microscópica vida que queda, resiste a la muerte. Este es un sistema

que recapitula los capítulos iniciales de la vida en el planeta, como una especie de resumen de los inicios que se acerca hacia su fin. En esta parte del desierto de Atacama llueve rara vez, lo que hace que la evaporación sea un proceso continuo y negativo: se pierde más agua de la que ingresa, haciendo de este ecosistema un lugar extremadamente frágil, donde cada pequeño cambio es muy notorio. Esta fragilidad permite imaginar la desaparición futura de otros cuerpos acuáticos progresivamente afectados por procesos de hiper-salinización de las aguas y por evaporación. En lo que viene, mostraremos como hemos sido afectados fuertemente al relacionarnos con el salar y su vida evolutiva de tiempo-profundo, cultivando así un tipo de afecto de tiempo-profundo que tiende a quedar separado de los modos humanos en los que se experiencia el tiempo (Chakrabarty 2021).

Apenas habíamos llegado al salar, en una mañana de Mayo de 2017, Cristóbal notó con sorpresa como Cristina y el equipo de microbiólogos -que conocían ya muy bien este salar- se sintieron emocionalmente impactados por las transformaciones visibles del salar y sus alrededores: la compañía minera que trabajaba en el área había construido una red de señales con información pública sobre la vida microbiana del salar, además de una plataforma de acceso que permitía a posibles turistas acercarse a las aguas superficiales del salar.

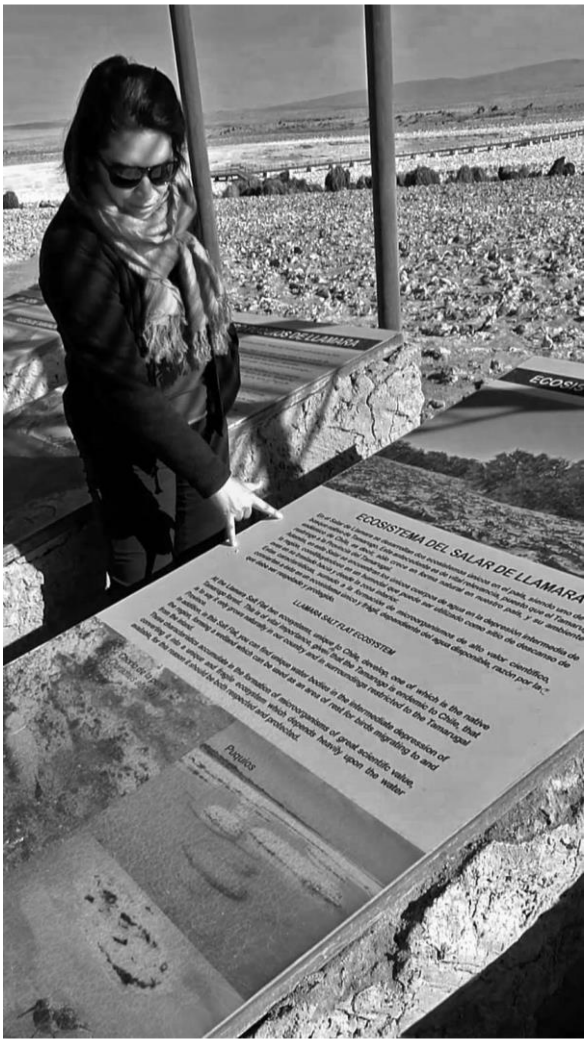

(Figura 6) Cristina leyendo las señales con información sobre microorganismos del Salar

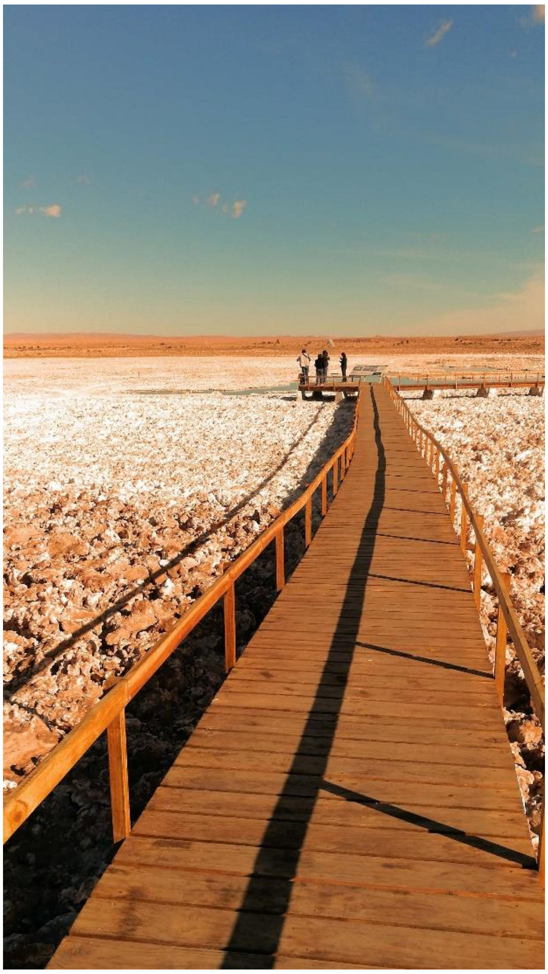

(Figura 7) Plataforma de acceso a las aguas del salar construida por la empresa minera (Foto: Cristóbal Bonelli).

“Esto es una aberración! Esto es una aberración!,”, fue la frase repetida una y otra vez por distintos miembros del equipo enfrentados a estas transformaciones con sus señaléticas y plataformas. Cristina, mientras leía las nuevas señales, con cierto sarcasmo e irritación enfatizaba lo absurdo que era usar la palabra ‘conservación’ en un lugar que estaba siendo destinado a actividades mineras que explotaban las aguas del salar y del acuífero.

Cuando nos acercamos caminando por la plataforma hacia las pozas del salar, pudimos ver información específica sobre los microrganismos contenida en la señalética que era parte del 'levantamiento de línea de base' requerido por la ley chilena medioambiental actual. Esta información, a pesar de la aberración que vivíamos, fue considerada importante por los microbiólogos, ya que en general, la ley solo contemplaba a la flora y la fauna, no a los microorganismos. Sin embargo, esa información no era suficiente para compensar la rabia que producía el constatar, una y otra vez, que los procedimientos para efectuar estudios de impacto ambiental eran totalmente ineficaces: en general, el *modus operandus* -comentaban varios miembros del equipo- es que los estudios de impacto ambiental sean hechos por consultoras pagadas y elegidas por las mismas mineras.[18]

Cristina y su equipo se sentían tristes, enojadas y frustradas -sentimientos que Cristóbal comenzó a compartir a medida que se involucraba en lo que estaba pasando y comenzaba a comprender la importancia de aquello de lo que estaba siendo testigo. El equipo veía estos cambios cosméticos como violentas intervenciones en la vida microbiana del salar, considerándolos como una estrategia de la compañía minera para decorar superficialmente los daños ambientales que había producido. Para empeorar las cosas, cuando nos acercamos a las aguas del salar, notamos que el salar tenía menos agua que de costumbre. La intensidad de nuestro sentimiento de aberración creció con mayor fuerza cuando estuvimos aún más cerca de un pequeño palito rojo y blanco usado para medir el nivel de las aguas, artefacto que usualmente es fotografiado por los administradores de la superintendencia del medio ambiente que fiscalizan.

[18] Para profundizar el modo en como los Estudios de Impacto Ambiental en el contexto chileno tienden a normalizar la situación actual que analizan, borrando de esta manera la memoria ambiental y devastación previa, ver Barandiarán 2020.

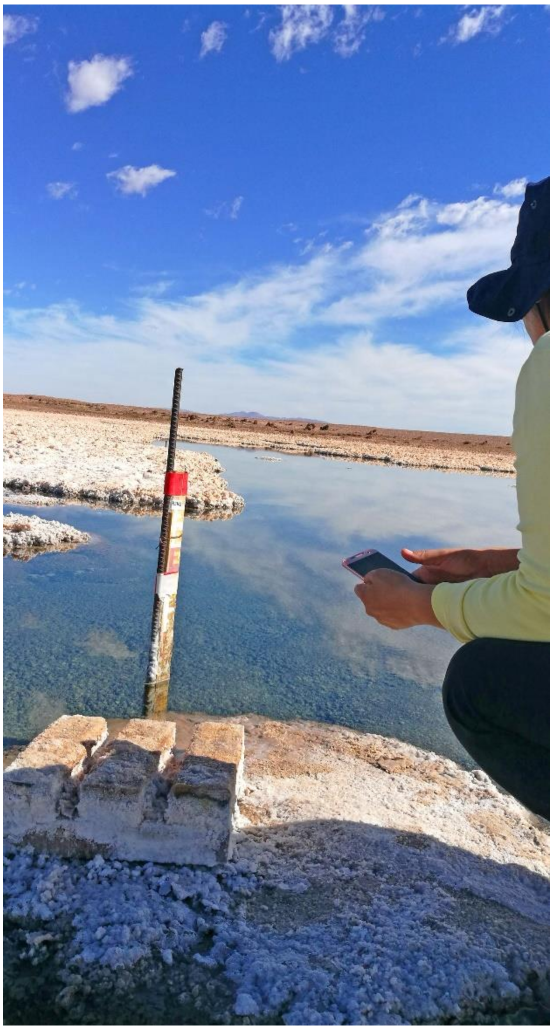

(Figure 8). Palito usado para medir el nivel de las aguas (Foto. Cristóbal Bonelli).

Una de las microbiólogas explicó:

> ‘Como hubo afectación del salar, el Consejo Regional de Tarapacá y la comunidad les puso una demanda (en contra la minera) como respuesta a los impactos de su operar en el salar. Entonces ahora por ley la minera tiene la obligación de mantener el nivel del agua, de manera tal que se conserven los *puquios*, que son los ojos de agua que tiene el salar. La minera quiere que los *puquios* estén siempre sumergidos porque si, de repente, chupan toda el agua, se seca todo… Se puede ver cuál fue el nivel de la poza, si te fijas hay sal acá, y acá esta blanquito, el nivel del agua llegó hasta acá e inundó esta zona (…) alguien se para acá, pero esta marca es fresca porque tiene sal, y luego la sal se cae; se subieron acá (*el bloque*) porque esto estaba inundado… O sea acá vienen a fiscalizar que realmente se esté inyectando agua, que el nivel del agua está ok (…) Todos estos procesos se hacen de noche; ellos extraen agua de noche, miden un par de veces, sacan la foto para mantener registro de que efectivamente se está haciendo por si los llegaran a controlar, y si baja mucho (el nivel de agua), abren las llaves de unas válvulas, de unas mangueras negras grandes y les inyectan agua dulce... es como si tuvieran una pecera con peces de mar y si se te empieza a evaporar el agua le empiezas a echar agua dulce de la llave, y esto es un agua híper salina, y obvio, los peces no van a responder bien y se te van a empezar a morir. Todos los microorganismos están adaptados para las aguas híper salinas!’

La intensidad de la aberración, sin embargo, solo alcanzó su clímax cuando encontramos una parte de un tapete microbiano, la forma más visible y colorida de la vida microbiana, flotando en las aguas.

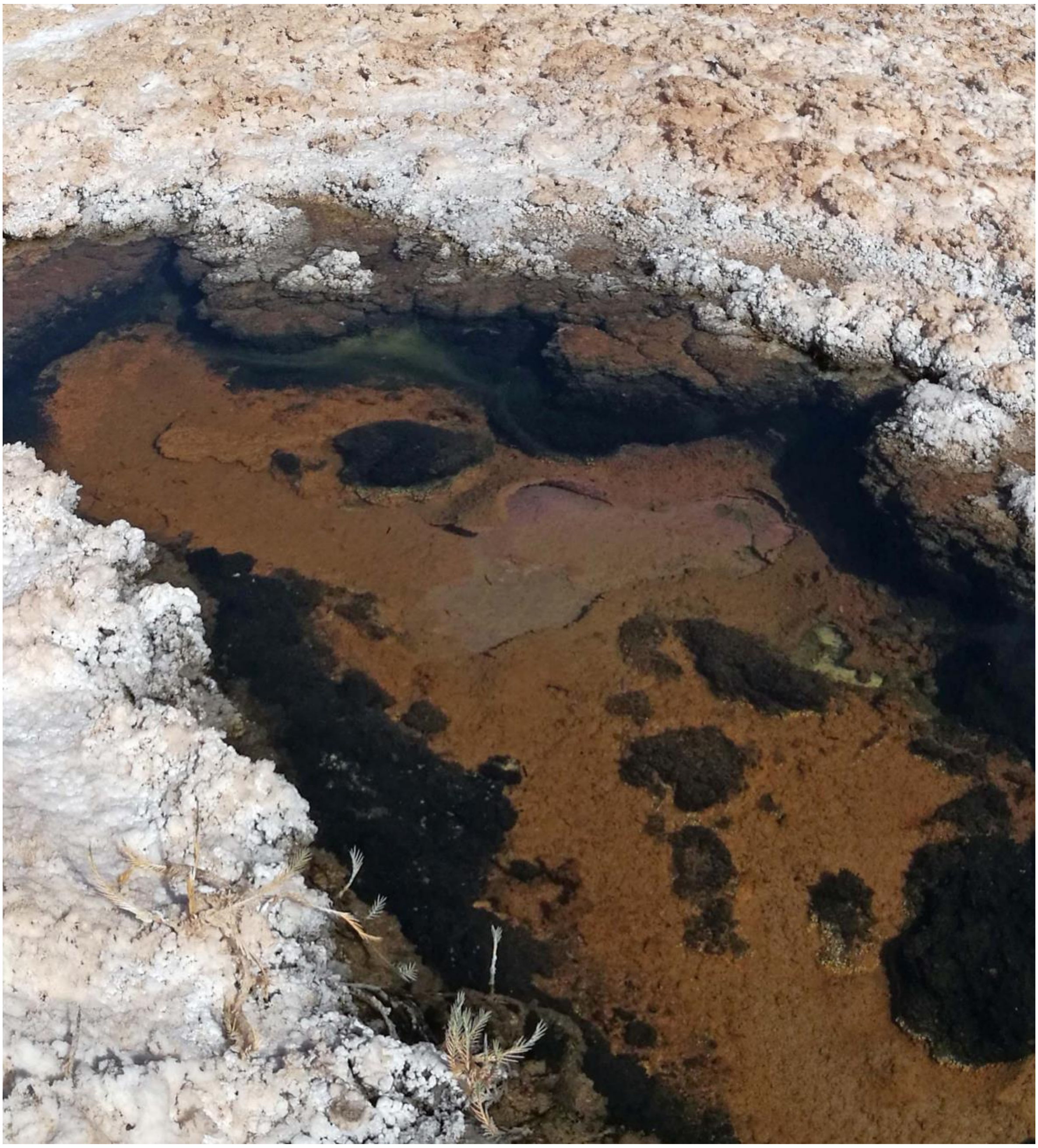

(Figura 9). Tapete microbiano flotando en las aguas del salar (Foto: Cristóbal Bonelli).

Este hecho alarmó aún más a un equipo de microbiólogos ya evidentemente sobrepasados con la situación. Para los ojos entrenados en microbiología de salares, el pedazo de tapete desprendido era la expresión de la muerte de una comunidad única de microrganismos, muerte que había sido producida por la inyección de agua dulce en un ecosistema altamente salino, donde habrían podido encontrarse bacterias aparecidas hace más de 2500 millones de años atrás (Gutiérrez-Preciado et al. 2018). Después de medir la conductividad eléctrica de las aguas, el equipo descubrió un cambio significativo en la salinidad de estos cuerpos de agua.

Debido a que la vida microbiana está adaptada a altos niveles de salinidad, un cambio repentino en los niveles de salinidad altera la estructura de los tapetes microbianos, quebrando las relaciones celulares y estructurales entre comunidades.

Al pasar de las horas, y de la las tristes y coléricas conversaciones, estos cambios cosméticos del salar y los micro-desastres de los cuales estábamos siendo testigos, progresivamente comenzaron a afectar, con mayor intensidad, a Cristóbal. Originalmente formado como psicólogo, y más tarde como antropólogo, Cristóbal había cultivado una sensibilidad especial respecto a eventos humanos y sus experiencias históricas del tiempo. En su práctica clínica, Cristóbal se había acostumbrado a escuchar, y ser afectado por, las biografías de individuos y familias con distintas historias de sufrimiento. Además, la investigación etnográfica más reciente de Cristóbal se había concentrado en estudiar territorios chilenos profundamente afectados por violencia de estado, realizando un gran esfuerzo en intentar conceptualizar y dar cuenta de las presencias de la ausencia; intentando comprender como algunas presencias ausentes que aparecen en el trabajo etnográfico -como las presencias de aquellos que habían sido asesinados en el lugar donde se construyó el memorial de Antofagasta- tenían la capacidad de afectar a los etnógrafos -y a sus conocimientos disciplinados- con sus fuerzas espectrales (Bonelli 2019; Bonelli y Poirot 2020).

La experiencia afectiva en Llamara inesperadamente despertó en Cristóbal su compromiso por pensar cómo temporalidades múltiples perseveran en su existencia, haciendo más complejos nuestros horizontes temporales. Sin embargo, las emociones gatilladas por la destrucción de las ecologías comprometidas en el tapete microbiano, estaban relacionadas a un afecto que excedía esos afectos propios de las temporalidades humanas. Esos afectos, de hecho, habían sido provocados por el tiempo-profundo de los salares y sus ecologías microbianas amenazadas por la actividad extractivista. Inesperadamente, la aberración sentida por los microbiólogos, y progresivamente por Cristóbal también, se transformaba en un tipo de afecto que conectaba experiencialmente las temporalidades de la evolución biológica con el tiempo histórico humano.

Después de un par de horas de conversación, en las que el equipo de microbiólogas compartía sus frustraciones, Cristina dijo, con un aire jocoso: ‘Ok, listo, ya nos hemos desahogado! Y hemos hecho psicoterapia con Cristóbal! Gracias Cristóbal! Todo este asunto es muy, pero muy frustrante, y nosotros no podemos hablar sobre ellos en nuestros trabajos científicos; nosotros no estamos entrenados para ello’.[19]

[19] Para un trabajo que analiza como la escritura científica obscurece a los escritores humanos como agentes activos interesados en discutir la ‘naturaleza’ como existiendo en un modo ‘allá fuera’, ver Law 2015.

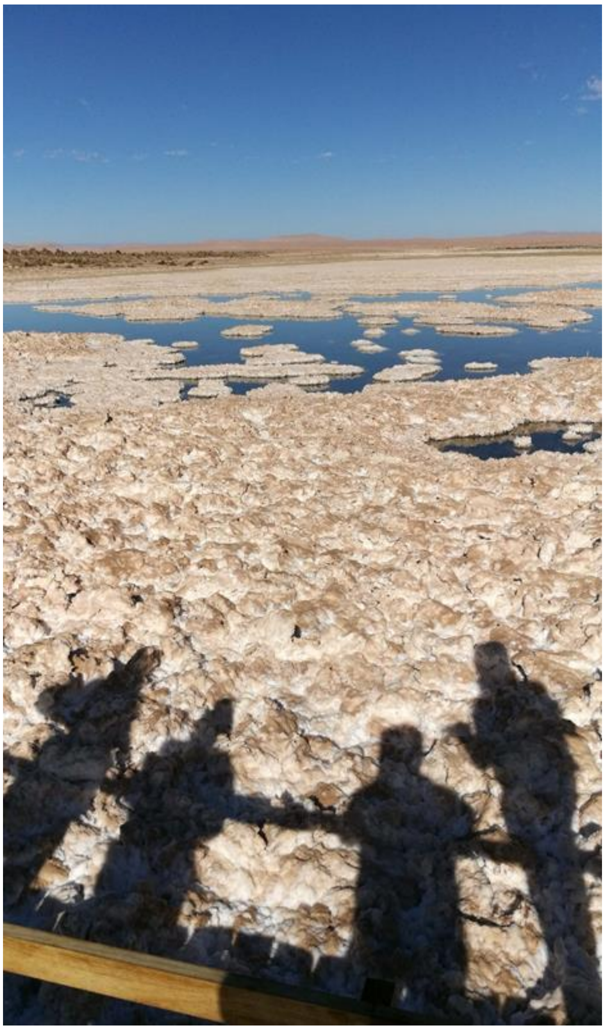

(Figure 10). Una conversación ‘terapéutica’ en el salar (Foto: Cristóbal Bonelli)

Cuatro años más tarde, sin embargo, nos hemos dado cuenta que esta broma de Cristina (‘hemos hecho psicoterapia!’), expresaba de alguna manera lo que hemos llegado a pensar como un *afecto de tiempo-profundo,* un tipo de afecto que conecta el tiempo histórico con las escalas temporales del tiempo evolutivo biológico, un tipo de afecto que, además, cuestiona fuertemente cierto tipo de naturalismo patriarcal que prohíbe dar espacio a las emociones dentro de sus descripciones científicas. El ser testigos de cómo el archivo microbiano de tiempo-profundo sobre el Gran Evento de Oxigenación estaba siendo alterado por la actividad extractivista nos invitaba no solamente a repensar la psicoterapia -al traer el tiempo de la evolución biológica a la vida cotidiana!-, sino que también nos obligaba a repensar la reparación ecológica desde temporalidades de procesos planetarios que excedían al tiempo histórico humano.

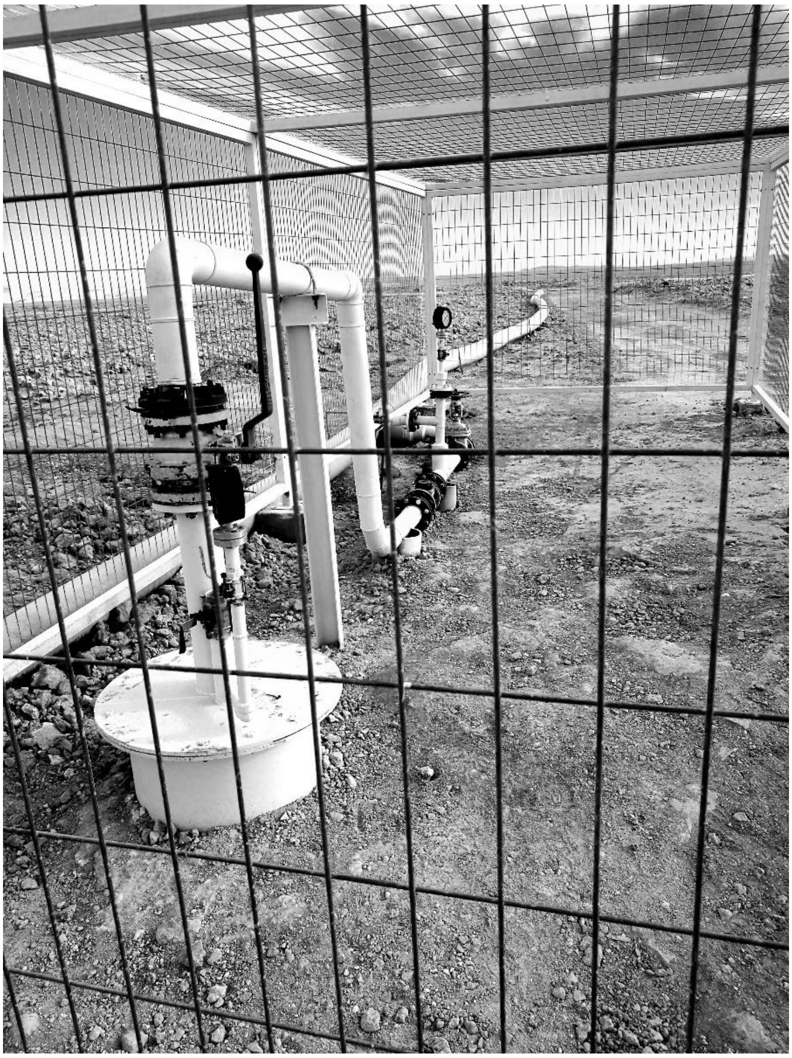

(Figure 11). Tuberías mineras en el salar de Llamara (foto: Cristóbal Bonelli)

Pesquisas preliminares indican que las aguas del Salar de Llamara son extraídas y transportadas unos 8 kilómetros al norte del salar, donde son usadas para disolver rocas de ‘caliche’, de las cuales se producen tipos diferentes de nitratos que son usados para hacer sales solares, y en definitiva, ‘energía limpia’. Estas sales, a su vez, son puestas a trabajar para el almacenamiento termal y la transferencia de calor en plantas termosolares de concentración (CSP). De hecho, en el medio del Desierto de Atacama, está comenzando sus operaciones la planta concentradora termosolar de Cerro Dominador, la más grande de Latinoamérica y que tiene una capacidad de 110 KMW y 17,5 horas de almacenamiento termal. Su torre principal tiene una altura de 220 metros, por lo cual es posible verla desde distintos puntos del desierto. Las sales necesarias para su funcionamiento son principalmente nitrato de potasio y cloruro de sodio, sales abundantes en el Desierto. Las sales de potasio se obtienen desde las salmueras del Salar de Atacama (donde además se encuentras las empresas de litio más grandes del planeta); y las sales de nitrato de sodio (salitre) son obtenidas desde los depósitos de caliche

de la pampa a través de un proceso que utiliza el agua del Salar de Llamará y del acuífero de la Pampa del Tamarugal.

No sabemos con total exactitud que uso se hace de las aguas extraídas del salar de Llamara, la que fue reemplazada por agua dulce. Lo que nos interesa mostrar acá al traer el evento del que fuimos testigos en Llamara es como los salares del norte de Chile están siendo fuertemente alterados por la extracción de aguas subterráneas, y por supuestamente 'pequeños' cambios en estos cuerpos de agua: en el caso de Llamara, el poner las aguas del salar a trabajar, derivó en una aniquilación biológica. Pero, ¿Qué implica esta aniquilación biológica por la imaginación de tiempo-profundo de la microbiología?

4. Tapetes microbianos: centinelas ancestrales del sol

Para la imaginación microbiológica, los microorganismos son los primeros organismos que existieron en la Tierra, y la evolución de ellos permitió la oxigenación del planeta hace 2500 millones de años atrás, y al mismo tiempo, causó la explosión de la biodiversidad. Al oxigenarse la atmósfera de la Tierra primitiva, cambiaron las condiciones físicas y químicas prevalentes, abriendo así una ventana de oportunidad para el cambio, para la diversificación.

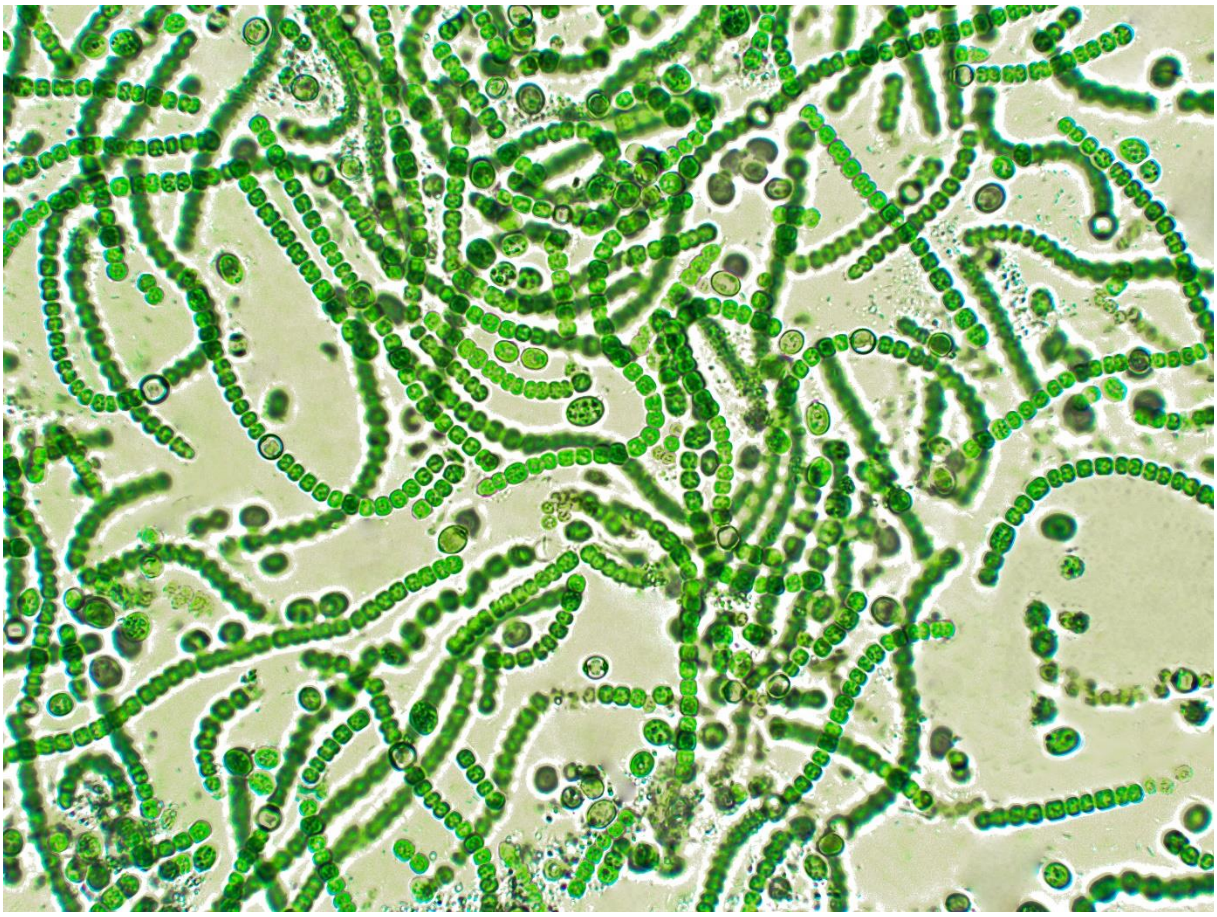

(Figure 12). Cianobacterias en el microscopio (Foto: Elif Bayraktar/ iStock)

Miremos por un momento a una cianobacteria bajo el lente del microscopio: las células bacterianas están formadas por diferentes moléculas que se basan en carbono (proteínas, azúcares, lípidos, ácidos nucleícos). A través del uso de la radiación solar, estos organismos fotosintéticos transforman el dióxido de carbono atmosférico en moléculas orgánicas (a través de un proceso llamado ‘fijación de dióxido de carbono’). De hecho, en biología descarbonización implica el proceso de *fijar el dióxido de carbono* desde la atmósfera hacia dentro de las células, para que de esta manera ser utilizado con otros propósitos. Además, algunos microorganismos en ambientes extremos pueden usar el dióxido de carbono para obtener energía (Cordero et al. 2019). Células microbianas originales (microorganismos) usaron compuestos inorgánicos para obtener energía. Por lo tanto, ellas emergieron como un nexo esencial entre el mundo inorgánico y el mundo organísmico emergente.

Los microorganismos pueden obtener su energía y carbono desde fuentes orgánicas o inorgánicas. Los metabolismos más ancestrales fueron inorgánicos, y operaron en una conexión íntima con el entorno. Se presume que el ancestro de la vida celular en la Tierra (*Last Universal Common Ancestor*) fue un microorganismo anaeróbico, fijador de dióxido de carbono, fijador de nitrógeno, dependiente de hidrógeno y termófilo (Weiss et al. 2016). La vida misma, de hecho, comenzó como una complejización del engranaje de lo inorgánico.

En términos generales, los microorganismos usan y degradan diferentes compuestos orgánicos e inorgánicos porque ellos contienen enzimas específicas para esas funciones. A pesar de su pequeño tamaño y su aparente invisibilidad, los microorganismos son comunidades vivas ubicuas, diversas y abundantes que se relacionan con todo tipo de organismos; los microorganismos son la red invisible y silenciosa de la biosfera.

A pesar de su aparente invisibilidad, y aunque los microorganismos necesitan de los microbiólogos para hacerlos visibles, ellos pueden ser detectados en manera visual en algunos ambientes. Por ejemplo, algunas áreas dispersas del Salar del Huasco son una congregación de microalgas y cianobacterias, áreas que son verdes porque contienen un pigmento llamado clorofila, crucial para la fotosíntesis.[20] En los océanos, la clorofila es parte de organismos

[20] Para un relato antropológico muy inspirador sobre la fotosíntesis, ver Myers 2016.

tales como las cianobacterias, y las micro y macroalgas. Estos organismos son responsables del 40% de la fijación del carbón en la Tierra.

Los microorganismos están constantemente interactuando con el carbono en distintos niveles y lugares del planeta. Incluso los virus, no reconocidos abiertamente como seres vivos, son considerados la 'bomba de carbono' del océano (Staff 2020), ya que al infectar las abundantes células microbianas existentes en el océano (fitoplancton, bacterias y otros) liberan carbono. Este carbono, a su vez, es utilizado directamente por otros organismos (zooplancton), los cuales, al morir, se depositan en el sedimento siendo degradados por otros microorganismos. Recientemente algunos estudios han reportado que las cifras de fijación de carbono estarían subestimadas al no considerar aspectos oceanográficos como la profundidad de la penetración de la luz (Buesseler et al. 2020).

Teniendo en cuenta lo dicho sobre los microorganismos, podemos ahora apreciar mejor a un tapete microbiano en condiciones típicas.

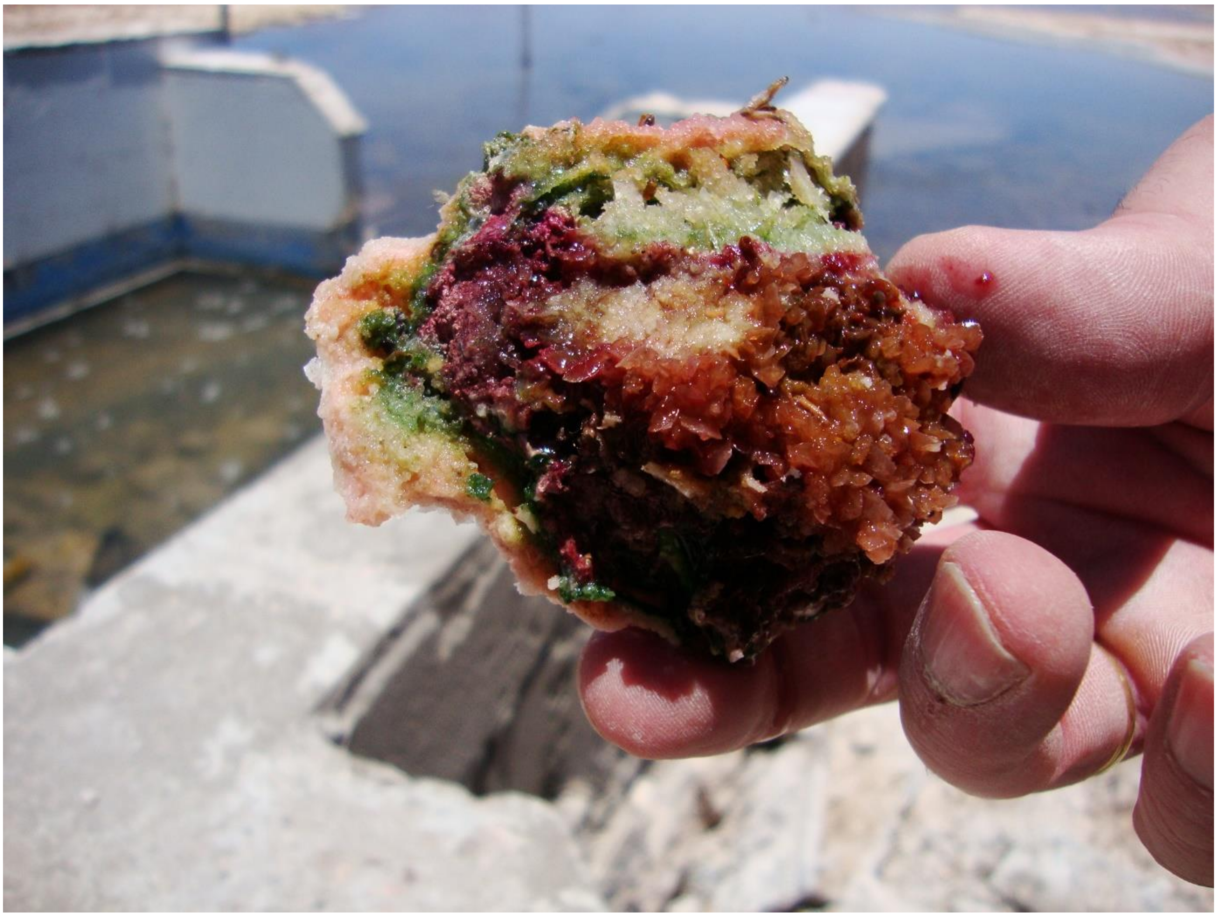

(Figura 13). Colorido tapete microbiano (Foto: Cristina Dorador).

Los tapetes microbianos son estructuras coloridas localizadas bajo la extensión de la blanca costra de sal de los salares, como el salar de Llamara. Cada color del tapete microbiano indica un tipo diferente de comunidad microbiana que se encuentra realizando una dinámica de intracción física, química y genética. Rosado, naranjo, verde, purpura, café y negro, son cada uno, colores que representan pigmentos bacterianos de grupos diferentes: *Roseobacter*, *Sphingomonas*, *Leptolyngbya*, *Chromatium*, *Bacillus*, y *Desulfovibrio*, respectivamente. Cada una de estas capas captura porciones diferentes del espectro solar, realizando así una distribución *justa* de la energía solar. Diferentes longitudes de onda de la radiación solar son detectadas por distintos pigmentos y, consecuentemente, por distintos microorganismos. La diversidad de microorganismos está adaptada a la diversidad del espectro solar. Cada comunidad fotosintética fija el dióxido de carbono, y lo convierte en materia orgánica. A su vez, las moléculas orgánicas son fuente de energía para otros microorganismos. Los microorganismos son fuente de alimento para zooplankton, insectos y protistas. Esta cadena continua, y armónica, de transformación del carbono, ocurre incluso bajo condiciones extremas. En conclusión, cada comunidad microbiana de un tapete microbiano alberga diferentes poblaciones y especies microbianas, cada cual poseedora de una singularidad única y compleja que existe en continuidad con el ambiente; ellas modifican el ambiente y el ambiente las modifica a ellas. Ellas son un forma compleja de heterogeneidad ontológicamente indisociable.

En los tapetes microbianos, los microorganismos exhiben visualmente una clara estratificación vertical basada en los colores diferentes de las capas; sin embargo, existen invisibles conexiones entre las capas mediadas por compuestos químicos, electrones, virus, ADN, gases, y otras moléculas orgánicas. En la superficie, los microorganismos están expuestos directamente a la alta radiación solar, y en conjunto con las sales, ellos forman un escudo efectivo que les permite también a otras bacterias realizar una singular utilización de la radiación solar. Los tapetes microbianos, así, son los centinelas ancestrales del sol. Por debajo de esta capa microbiana, cianobacterias realizan fotosíntesis, produciendo así grandes concentraciones de oxígeno que contribuyen al funcionamiento de otros organismos. Esta vida microbiana no sería posible, como hemos ya mencionado, sino fuese por la presencia del agua. En ecosistemas secos y áridos como los salares, el agua es escasa y en este sentido puede llegar a ser un factor limitante para el desarrollo de la vida. El agua *con* las sales *es* la vida microbiana. Por ejemplo, las salmueras -agua con altas concentraciones de sales, y desde la cual el litio es extraído-, contienen una gran diversidad de bacterias, arqueas, virus, y

células eucariotas (Cubillos et al. 2018). En este contexto, el tapete microbiano en conjunto con el agua y las condiciones ambientales específicas del desierto de Atacama, forman un ecosistema espacio-temporal dinámico, donde las modificaciones de uno de estos factores, como hemos visto en el caso del salar de Llamara, puede gatillar la destrucción de ecologías microbianas, de sus intracciones de tiempo profundo y finalmente provocar la pérdida de biodiversidad microbiana.

5. Hacia un modo de sobrevivencia holobióntico

Los eventos que vivimos en el Salar de Llamara muestran como la intimidad de tiempo-profundo de las ecologías microbianas es alterada por la actividad minera. Estos eventos muestran como la actividad extractiva destruye la intimidad de tiempo-profundo inherente a los salares y sus ecologías microbianas. De hecho, desde la perspectiva de la imaginación microbiológica, el salar de Llamara es un archivo que contiene la memoria de eventos tempranos de la Tierra. Sus tapetes microbianos recapitulan el Gran Evento de Oxigenación (Gutiérrez-Preciado et al. 2018). Sin embargo, la memoria microbiana colectiva y de tiempo-profundo presente en un pequeño charco en el medio del desierto de Atacama está siendo borrada por las gramáticas separatistas propias del extractivismo, hecho que nos obliga a pensar con mayor detención cómo es posible sostener la *habitabilidad planetaria* y no la depredación de los recursos planetarios (Langmuir and Broecker 2012). Considerando lo anterior, estamos convencidos que la desaparición de los hábitat microbianos del desierto debieran ser considerada como micro-desastres, como un tipo de desastre a nivel microbiano que afecta y amenaza los vestigios pasados del planeta Tierra, y una de las pocas conexiones -que son también conexiones afectivas!- que quedan con *nuestros* orígenes. Aquí, estamos usando la palabra *nuestros* a propósito, porque nos referimos a un origen que nos incluye a nosotros -a Cristina, a Cristóbal, y también a ti, querido lector o lectora- dentro de una alianza de tiempo-profundo que involucra a una multitud de especies. De hecho, 'ellos', los microorganismos con sus intracciones, también comunican como un 'nosotros complejo': basta considerar que dentro del cuerpo de un adulto saludable 'el número de bacterias es de hecho muy parecido al número de células humanas' (Sender, Fuchs, and Milo 2016). Nuestros cuerpos humanos son comunidades de diferentes tipos de células: humanas, bacterianas, arqueanas, y hongos, células que a su vez se relacionan en modos complejos entre ellas. De hecho, *ser humano* implica *ser microorganísmico,* y envuelve 'las fusiones y mezclas de compañeros planetarios que nos han precedido en el microcosmos' (Margulis 1998, 12). Estamos hechos de bacterias y arqueas, del mismo modo en que lo estuvo la primera célula

eucariota. Diferentes especies son la expresión de ese pasado y presente de intracciones microbianas. Aspectos del funcionamiento de nuestro cerebro -y de nuestro mismo modo de pensar y generar conocimiento sobre microorganismos- se base en la generación bacteriana de neurotransmisores en nuestros intestinos. En definitiva, somo humanos porque al mismo tiempo somos bacteria y microorganismos.

'Nosotros' somos, de hecho, 'holobiontes'

> ensamblajes simbióticos…, más parecidos a nudos de relacionamientos intractivos en sistemas dinámicos complejos, que a entidades de una biología hecha de unidades pre-existentes y delimitadas (genes, células, organismos, etc) en interacciones que solo puede ser concebidas como competitivas o cooperativas. (Haraway 2016, 60).

Y al ser holobiontes, nosotros somos tiempo-profundo e historia-profunda, lo que implica, como Penny Harvey (2019) ha sugerido recientemente, comprender que 'la relación constitutiva y mutua entre humanos y no-humanos no está bajo el control humano, aun cuando la intención humana pareciera ser primordial' (Harvey 2019, 188). La industria minera, sin embargo, deshace esos tejidos intractivos constitutivos de las ecologías microbianas de los salares con los que nosotros somos y existimos en continuidad. Por lo tanto, la preservación y conservación de salares, y sus comunidades microbianas, necesita de una profunda crítica respecto a la producción de energía renovable (Jasanoff 2007; Boyer 2019; Cross 2019; Daggett 2019) y sus arreglos técnicos universalistas de 'talla única' y supuestamente sustentables (Hulme 2014; cf Nadim 2016) que dependen de minerales críticos como el litio que se encuentra en las antiguas salmueras de los salares. Como mínimo, el efecto de la producción de energía moderna y la minería de salares requiere que nos cuestionemos radicalmente las lógicas energéticas que operan a través de prácticas, gramáticas y lenguajes de desconexión que facilitan un lento e invisible avance destructivo del medio ambiente con el que somos (Nixon 2011). De hecho, compresiones microbiológicas sobre descarbonización (por ejemplo, la fijación microbiana de dióxido de carbono), están siendo silenciadas y amenazadas por otros modos de comprender la descarbonización dominados por la gramática extractivista y su ideal de 'escalabilidad'. Concretamente, la fijación microbiana de dióxido de carbono hace parte de un 'nosotros ambientalmente complejo', y como tal, es una aliada que necesita ser hecha pública y visible. De hecho, nuestros aliados son los microorganismos -los 'co-autores incontables de Gaia' (Stengers 2015)-, quienes, a pesar de las prácticas destructivas propias de aquella época geológica que podríamos pensar como el *extractoceno*, van 'a continuar participando en manera efectiva en el régimen de existencia de Gaia como planeta viviente' (Stengers 2015, 47). Considerando que los microorganismos sobrevivirán a nuestra posible extinción como especie humana,

creemos que estamos en un momento oportuno para visibilizar su invisibilidad, para aprender de ellos, para ser afectados por ellos y por su tiempo-profundo, el cuál es, simultáneamente, también nuestro propio tiempo.

Nuestro objetivo en este artículo ha sido ofrecer una experimentación conceptual capaz de generar nuevas posibilidades de comunicación e imaginación entre cuerpos afectados (Despret 2004; Morita and Susuki 2019); de hecho, nuestra colaboración ha intensificado nuestra capacidad para aprender a ser afectados por estas ecologías de tiempo-profundo. Al ofrecer este ejercicio narrativo que muestra como nuestros compromisos afectivos con los salares nos han ayudado a desarrollar un modo de pensar centrado en el planeta (Zalasiewicz 2020), hemos intentado descentrar concepciones humano-céntricas de la política (Deleuze 1968; Latour 1993; Bonelli 2016; de la Cadena 2019) y proponer una reconsideración radical de la misma humanidad -como Felix Guattari nos sugería en el epígrafe al inicio de este artículo. Nosotros pensamos que hacer esto no implica proponer que entendemos a los salares, y a sus ecologías microbianas de tiempo-profundo, como totalmente retiradas de la experiencia humana (Harman 2018). Lo que hemos querido mostrar, en realidad, es como nuestras propias maneras de relacionarnos con los salares han intensificado nuestra modo de entender a lo humano ‘como el trabajo de miles de millones de años de interacción entre microbios altamente sensibles’ (Margulis 1998, 4).

Finalmente, en el contexto de este número especial interesado en pensar la extinción, quisiéramos concluir afirmando que pensar la extinción implica, impajaritablemente, responder al problema sobre cómo conceptualizar la unidad de sobrevivencia en modo apropiado. Nosotros estamos convencidos que nuestras preocupaciones con la extinción, y al mismo tiempo, con la justicia, no pueden ser preocupaciones sobre humanos que se consideran como existiendo en manera separada de las ecologías de tiempo-profundo y de sus afectos de tiempo-profundo. Y es por este motivo que nosotros estamos trabajando arduamente para crear urgentes alianzas experimentales, capaces de pensar, sentir, actuar y proponer un modo de sobrevivencia holobióntico.

Agradecimientos

Agradecemos a Bernardita Valenzuela, Daniela Meneses, Drina Vejar, and Irma Vila por ser fuente de inspiración constante y por el apoyo de nuestros terrenos en los salares. Queremos agradecer también a Marina Weinberg, Jamie Pitts, Sebastián Ureta, Gabrielle Hetch, Edward Simpson, Penny Harvey, Arianna Injeian and Michelle Geraerts por haber generosamente comentado versiones anteriores de este artículo. Agradecemos además a los revisores anónimos de Tapuya, y a los editores de este número especial, quienes nos ayudaron a clarificar el texto con sus valiosas sugerencias. Versiones breves de este trabajo fueron presentadas en la conferencia Internacional KCE 2017, en Santiago de Chile; en la conferencia anual de 4S 2019 en Nueva Orleans, y en la conferencia RAI2020 Antropología y Geografía: Diálogos pasados, presentes y futuros, organizada por el Royal Anthropological Institute, British Museum, SOAS, RGS.

Financiamiento

Este trabajo contó con el apoyo del European Research Council (ERC) y el programa de investigación e innovación de la comunidad Europea Horizon2020 (grant agreement No. [853133]). Parte del terreno de Cristóbal tuvo el apoyo de su beca Marie Curie Global Fellowship 'Invisible Waters' (n. 706346), financiada por Horizon 2020, y apoyada por CIGIDEN, ANID/FONDAP/15110017 y CIIR, ANID/FONDAP/1511006.

Nota sobre los autores

Cristóbal Bonelli es psicólogo, psicoterapeuta y profesor asociado de Antropología en el departamento de Antropología de la Universidad de Amsterdam. Es investigador principal del proyecto ERC 'Mundos de Litio' (https://worldsoflithium.eu/). Cristóbal también colabora con el Centro de Investigación para la Gestión Integrada del Riesgo de Desastres (CIGIDEN) y el Centro de Estudios Interculturales e Indígenas (CIIR).

Cristina Dorador es microbióloga, experta en ecología microbiana, limnología y geomicrobiología. Cristina es profesora asociada en el departamento de Biotecnología de la Facultad de Ciencias Naturales y Marinas de la universidad de Antofagasta. Desde Julio 2021, Cristina es una de los 155 personas elegidas democráticamente para escribir la nueva constitución chilena dentro la Convención Constitucional.

REFERENCIAS

Ampuero, P. 2016. "Diplomacia En Transición. La República Popular China Frente a La Dictadura
Cívico-Militar En Chile." Estudios Políticos (Medellín) 49 (2016): 35–54.

Agusdinata, D. B., W. Liu, H. Eakin, and H. Romero. 2018. "Socio-environmental Impacts of Lithium
Mineral Extraction: Towards a Research Agenda." Environ. Res. Lett 13: 123001.
https://iopscience.iop.org/article/10.1088/1748-9326/aae9b1.

Babidge, S. 2018. "Sustaining Ignorance: the Uncertainties of Groundwater and its Extraction in the Salar de Atacama, Northern Chile." Journal of the Royal Anthropological Institute 25 (1): 83–102. doi:10.1111/1467-9655.12965.

Barandiarán, J. 2020. "Documenting Rubble to Shift Baselines: Environmental Assessments and Damaged Glaciers in Chile." Environment and Planning E: Nature and Space 3 (1): 58–75. doi:10.1177/2514848619873317.

Barad, K. 2007. Meeting the Universe Halfway: Quantum Physics and the Entanglement of Matter and Meaning. Second Printing ed. Durham: Duke University Press Books.

Bateson, G. 1972. Steps to an Ecology of Mind: Collected Essays in Anthropology, Psychiatry, Evolution, and Epistemology. Chicago: University of Chicago Press.

Bauer, C. 2015. "Water Conflicts and Entrenched Governance Problems in Chile's Market Model." Water Alternatives 8 (2): 147–172. http://www.water-alternatives.org/.

Bebbington, A. 2015. "Political Ecologies of Resource Extraction: Agendas Pendientes." ERLACS 0(100): 85–98. doi:10.18352/erlacs.10121.

Blaser, M., and M. de la Cadena. 2018. A World of Many Worlds. Durham: Duke University Press.

Bonelli, C. 2016. "Palabras de piedra, materiales proféticos y políticas del dónde." Antípoda 26:19–43.

Bonelli, C. 2019. "Spectral Forces, Time and Excess in Southern Chile." In Chapter Within the Book The World Multiple Everyday Politics of Knowing and Generating Entangled Worlds, edited by K. Omura, G. Otsuki, S. Satsuka, and A. Morita, 123–139. New York: Routledge Advances in Sociology.

Bonelli, C., and L. Poirot. 2020. "Secretos de luz: apuntes para una antropología expuesta." En revista Antípoda. Revista de Antropología y Arqueología 41 (4): 175–201.

Boyer, D. 2019. Energopolitics: Wind and Power in the Anthropocene. Illustrated ed. Durham: Duke University Press Books.

Buesseler, K. O., P. W. Boyd, E. E. Black, and D. A. Siegel. 2020. “Metrics That Matter for Assessing the Ocean Biological Carbon Pump.” Proceedings of the National Academy of Sciences 117 (18): 9679–9687. doi:10.1073/pnas.1918114117.

Castro, L. C. 2020. El bosque de la Pampa del Tamarugal y la industria salitrera: el problema de la deforestación, los proyectos para su manejo sustentable y el debate político (Tarapacá, Perú-Chile 1829-1941) | Castro Castro | Scripta Nova. Revista Electrónica de Geografía y Ciencias Sociales. RCUB. https://revistes.ub.edu/index.php/ScriptaNova/article/view/26507.

Chakrabarty, D. 2020. World-Making, “Mass” Poverty, and the Problem of Scale. Journal #114 December 2020 - e-Flux. https://www.e-flux.com/journal/114/366191/world-making-masspoverty-and-the-problem-of-scale/.

Chakrabarty, D. 2021. The Climate of History in a Planetary Age. Chicago: University of Chicago Press.

Cordero, P. R. F., K. Bayly, P. Man Leung, C. Huang, Z. F. Islam, R. B. Schittenhelm, G. M. King, and C. Greening. 2019. “Atmospheric Carbon Monoxide Oxidation is a Widespread Mechanism Supporting Microbial Survival.” The ISME Journal 13 (11): 2868–2881. doi:10.1038/s41396-019-0479-8.

Cross, J. 2019. The Solar Good: Energy Ethics in Poor Markets.” Journal of the Royal Anthropological Institute 25 (S1): 47–66. doi:10.1111/1467-9655.13014.

Cubillos, C. F., P. Aguilar, M. Grágeda, and C. Dorador. 2018. “Microbial Communities from the World’s Largest Lithium Reserve, Salar de Atacama, Chile: Life at High LiCl Concentrations.”Journal of Geophysical Research: Biogeosciences 123 (12): 3668–3681. doi:10.1029/2018jg004621.

Daggett, C. N. 2019. The Birth of Energy: Fossil Fuels, Thermodynamics, and the Politics of Work (Elements). Durham: Duke University Press Books.

Davies, Jeremy. 2016. The Birth of the Anthropocene. Oakland: University of California Press. Accessed June 9, 2021. http://www.jstor.org/stable/10.1525/j.ctv1xxs47.

de la Bellacasa, Maria Puig. 2017. Matters of Care: Speculative Ethics in More Than Human Worlds. Minneapolis: University of Minnesota Press.

de la Cadena, M. 2019. “Uncommoning Nature: Stories from the Anthropo-Not-Seen’.” In Anthropos and the Material, edited by K. G. Nustad, C. Krohn-Hansen, and P. Harvey, 35–58. Durhman: Duke University Press Books.

Deleuze, Gilles. 1968. Spinoza et le problème de l’expression. Paris: Éditions de Minuit.

Demergasso, C., G. Chong, P. Galleguillos, L. Escudero, M. Martinez-Alonso, and I. Esteve. 2003. “Tapetes microbianos del Salar de Llamará, norte de Chile.” Revista Chilena de Historia Natural 76 (3): 485–499. doi:10.4067/s0716-078(2003000300012.

Despret, Vinciane. 2004. “The Body we Care for: Figures of Anthropo-zoo-Genesis.” Body & Society 10 (2–3): 111–134.

Dorador, Cristina. 2007. Microbial diversity in high altitude wetlands of the Chilean Altiplano: phylogeny, diversity and function. PhD Thesis, University of Kiel, Germany.

Guattari, F. 2000. The Three Ecologies (translated by Ian Pindar and Paul Sutton). Athlone Press, London and New Brunswick, NJ, 174 p.

Gudynas, E. 2010. “El Nuevo Extractivismo Progresist.” El Observador Del Obie 8: 1–10. http://cdi.mecon.gov.ar/bases/doc/obie/8.pdf.

Gudynas, E. 2018. “Extractivisms.” In Reframing Latin American Development, edited by R. Delgado, and R. Munck, 61–76. New York: Routledge Critical Development Studies.

Gutiérrez-Preciado, A., A. Saghaï, D. Moreira, Y. Zivanovic, P. Deschamps, and P. López-García. 2018.“Functional Shifts in Microbial Mats Recapitulate Early Earth Metabolic Transitions.” Nature Ecology & Evolution 2 (11): 1700–1708. doi:10.1038/s41559-018-0683-3.

Haraway, D. J. 2016. “Sympoiesis: Symbiogenesis and the Lively Arts of Staying with the Trouble.” In Staying with the Trouble: Making Kin in the Chthulucene (Experimental Futures) (p. Chapter 3), 58–98. Durham: Duke University Press Books.

Harman, G. 2018. Object-Oriented Ontology: A New Theory of Everything. London: Pelican.

Harvey, Penny. 2019. “6. Lithic Vitality: Human Entanglement with Nonorganic Matter.” In Anthropos and the Material, edited by Penny Harvey, Christian Krohn-Hansen, and Knut G. Nustad, 143–160. Durhman: Duke University Press.

Helmreich, S. 2014. “Homo Microbis: The Human Microbiome, Figural, Literal, Political.” Thresholds 42: 52–59.

Helmreich, S. 2016. New Geographies, 8: Island, edited by P. Pérez-Ramos, and D. Daou; Vol. 8 Harvard Graduate School of Design. https://www.gsd.harvard.edu/publication/island/

Hetch, G. 2018. “Interscalar Vehicles for an African Anthropocene: On Waste, Temporality, and Violence.” Cultural Anthropology 33 (1): 109–141.

Hulme, M. 2014. Can Science Fix Climate Change?: A Case Against Climate Engineering (New Human Frontiers). 1st ed. Cambridge: Polity Press.

Jasanoff, S. 2007. “Technologies of Humility.” Nature 450: 33. doi:10.1038/450033a.

Jerez, B., I. Garces, and R. Torres. 2021. “Lithium Extractivism and Water Injustices in the Salar de Atacama, Chile: The Colonial Shadow of Green Electromobility.” Political Geography 87: 102382.https://www.sciencedirect.com/science/article/abs/pii/S0962629821000421?via%3Dihub.

King, G. M. 2015. “Carbon Monoxide as a Metabolic Energy Source for Extremely Halophilic Microbes: Implications for Microbial Activity in Mars Regolith.” Proceedings of the National Academy of Sciences 112 (14): 4465–4470. doi:10.1073/pnas.1424989112.

Kleinman, A. 2012. “KAREN BARAD Intra-Actions.” Mousse Magazine 34), http://moussemagazine.it/product/mousse-34/.

Knowles, S. G. 2014. “Learning from Disaster?: The History of Technology and the Future of Disaster Research.” Technology and Culture 55 (4): 773–784. doi:10.1353/tech.2014.0110.
Köppel, J. 2020. "Lithium Transformations: An Unfinished Story." Transformations 33, 27–47.

Labban, M. 2014. “Deterritorializing Extraction: Bioaccumulation and the Planetary Mine.” Annals of the Association of American Geographers 104 (3): 560–576. doi:10.1080/00045608.2014.892360.

Langmuir, Charles H., and Wally Broecker. 2012. How to Build a Habitable Planet: The Story of Earth from the Big Bang to Humankind – Revised and Expanded Edition. REV – Revised ed. Princeton, NJ: Princeton University Press.

Latour, Bruno. 1993. We Have Never Been Modern. Nueva York: Harvester-Wheatsheaf.

Law, John. 2015. “What’s Wrong with a one-World World?” Distinktion: Journal of Social Theory 16 (1): 126–139. doi:10.1080/1600910X.2015.1020066.

Li, F. 2015. Unearthing Conflict: Corporate Mining, Activism, and Expertise in Peru. Durhman: Duke University Press Books.

Liu, W., D. B. Agusdinata, and S. W. Myint. 2019. “Spatiotemporal Patterns of Lithium Mining and Environmental Degradation in the Atacama Salt Flat.” Chile International Journal of Applied Earth Observation and Geoinformation 80: 145–156.

Margulis, L., and R. Fester. 1991. Symbiosis as a Source of Evolutionary Innovation: Speciation and Morphogenesis (The MIT Press). Cambridge, Mass: The MIT Press.

Margulis, L. 1998. Symbiotic Planet. A New Look at Evolution. New York: Basic Books.
Myers, Natasha. 2016. “Photosynthesis.” Theorizing the Contemporary, Fieldsights, January 21. https://culanth.org/fieldsights/photosynthesis

McFall-Ngai, M. 2017. “Noticing Microbial Worlds: the Postmodern Synthesis.” In Arts of Living on a Damaged Planet: Ghosts and Monsters of the Anthropocene, (3rd ed., p. Chapter 3) edited by A. L.Tsing, N. Bubandt, E. Gan, and H. Swanson, 51–70. Minneapolis: University of Minnesota Press.

Morita, Atsuro, and Wakana Susuki. 2019. “Being Affected by Sinking Deltas Changing Landscapes, Resilience, and Complex Adaptive Systems in the Scientific Story of the Anthropocene.” Current Anthropology 60 (20): S286–S295. doi:10.1086/702735.

Nadim, T. 2016. “Blind Regards: Troubling Data and Their Sentinels.” Big Data & Society 3 (2): 1–6. doi:10.1177/2053951716666301.

Nixon, R. 2011. Slow Violence and the Environmentalism of the Poor. Cambridge: Harvard University Press.

Pietrzyk, S., and B. Tora. 2018. “Trends in Global Copper Mining – a Review.” IOP Conference Series: Materials Science and Engineering 427: 1–11. doi:10.1088/1757-899x/427/1/012002.

Satsuka, S. 2015. Nature in Translation: Japanese Tourism Encounters the Canadian Rockies. Durham:Duke University Press Books.

Sender, R., S. Fuchs, and R. Milo. 2016. “Revised Estimates for the Number of Human and Bacteria Cells in the Body.” PLOS Biology 14 (8): e1002533. doi:10.1371/journal.pbio.1002533.

Staff, S. X. 2020. The Ocean’s “Biological Pump” Captures more Carbon than Expected. https://phys. org/news/2020-04-ocean-biological-captures-carbon.html.

Stengers, I. 2015. In Catastrophic Times: Resisting the Coming Barbarism. London: Open Humanities Press.

Stengers, I. 2018. “The Challenge of Ontological Politics.” In A World of Many Worlds, edited by M.Blaser, and D. M. Cadena. (Illustrated ed., pp. 83–111). Durhman: Duke University Press Books.

Stengers, I. 2020a. We Are Divided. Journal #114 December 2020 - e-Flux. https://www.e-flux.com/journal/114/366189/we-are-divided/.

Svampa, M. 2015. “Commodities Consensus: Neoextractivism and Enclosure of the Commons in Latin America.” South Atlantic Quarterly 114 (1): 65–82. doi:10.1215/00382876-2831290.

Tsing, A. L. 2016. The Mushroom at the End of the World: On the Possibility of Life in Capitalist Ruins. Illustrated ed. Princeton, NJ: Princeton University Press.

Viguier, B., H. Jourde, V. Leonardi, L. Daniele, C. Batiot-Guilhe, G. Favreau, and V. De Montety. 2019. Water Table Variations in the Hyperarid Atacama Desert: Role of the Increasing Groundwater Extraction in the pampa del tamarugal (Northern Chile).” Journal of Arid Environments 168: 9–16. doi:10.1016/j.jaridenv.2019.05.007.

Weinberg, M. 2021. “Cuerpos de cobre: Extractivismo en Chuquicamata, Chile.” The Journal of Latin American and Caribbean Anthropology 26 (2): 1–19.

Weiss, M. C., F. L. Sousa, N. Mrnjavac, S. Neukirchen, M. Roettger, S. Nelson-Sathi, and W. F. Martin. 2016. “The Physiology and Habitat of the Last Universal Common Ancestor.” Nature Microbiology 1 (9), n/a, doi:10.1038/nmicrobiol.2016.116.

Westermann, A. 2020. “Enrichment and Dilution in the Atacama Mining Desert. Writing History from an Earth-Centered Perspective.” Geschichte und Gesellschaft 46 (4): 634–661.

Yáñez, N., and R. Molina. 2011. Las aguas indígenas en Chile (Spanish Edition). Santiago: LOM Ediciones.

Zalasiewicz, Jan. 2020. “The Extraordinary Strata of the Anthropocene.” In Nature and Value, edited by Akeel Bilgrami, 29–45. New York: Columbia University Press
